# Potential Therapeutic Applications of Plant-Derived Alkaloids against Inflammatory and Neurodegenerative Diseases

**DOI:** 10.1155/2022/7299778

**Published:** 2022-03-09

**Authors:** Babita Aryal, Bimal Kumar Raut, Salyan Bhattarai, Sobika Bhandari, Parbati Tandan, Kabita Gyawali, Kabita Sharma, Deepa Ranabhat, Ranjita Thapa, Dipa Aryal, Atul Ojha, Hari Prasad Devkota, Niranjan Parajuli

**Affiliations:** ^1^Biological Chemistry Lab, Central Department of Chemistry, Tribhuvan University, Kirtipur, Kathmandu, Nepal; ^2^Meakins-Christie Laboratories, Department of Medicine, McGill University, Montreal, Quebec, Canada; ^3^Department of Chemistry and Chemical Biology, The University of New Mexico, Albuquerque, NM, USA; ^4^Graduate School of Pharmaceutical Sciences, Kumamoto University, 5-1 Oe-honmachi, Kumamoto 862-0973, Japan

## Abstract

Alkaloids are a type of natural compound possessing different pharmacological activities. Natural products, including alkaloids, which originate from plants, have emerged as potential protective agents against neurodegenerative disorders (NDDs) and chronic inflammations. A wide array of prescription drugs are used against these conditions, however, not free of limitations of potency, side effects, and intolerability. In the context of personalized medicine, further research on alkaloids to unravel novel therapeutic approaches in reducing complications is critical. In this review, a systematic survey was executed to collect the literature on alkaloids and their health complications, from which we found that majority of alkaloids exhibit anti-inflammatory action via nuclear factor-*κ*B and cyclooxygenase-2 (COX-2), and neuroprotective interaction through acetylcholinesterase (AChE), COX, and *β*-site amyloid precursor protein activity. *In silico* ADMET and ProTox-II-related descriptors were calculated to predict the pharmacological properties of 280 alkaloids isolated from traditional medicinal plants towards drug development. Out of which, eight alkaloids such as tetrahydropalmatine, berberine, tetrandrine, aloperine, sinomenine, oxymatrine, harmine, and galantamine are found to be optimal within the categorical range when compared to nicotine. These alkaloids could be exploited as starting materials for novel drug synthesis or, to a lesser extent, manage inflammation and neurodegenerative-related complications.

## 1. Introduction

Since ancient times, natural products have been utilized to treat a wide variety of health complications and have high therapeutic potential; varieties of plants containing bioactive metabolites are used to treat inflammation, neurodegenerative disorders, and correlated complications with good efficiency [1]. Among them, alkaloids are a part of chemical defense in plants, structurally varied category defend themselves chemically, structurally varied category of nitrogen-containing secondary metabolites with strong pharmacological effects, and account for 60% of plant-derived drugs [2]. Alkaloids are prevalent in several botanical families like Amaryllidaceae, Apocynaceae, Papaveraceae, Asteraceae, Solanaceae, Rutaceae, Fabaceae, and Rubiaceae [3].

In modern medicine, plant-derived alkaloids get much attention in a steady supply of medication to treat chronic diseases such as cancer, diabetes, and neurological disorders. They not only protect plants from herbivores but also curb fungal and bacterial infestation, which broadens their use in medicine and other fields [4]. Because of their actions, alkaloids have a wide variety of pharmacological appliances in the therapeutic area such as analgesic (e.g., morphine), antiasthmatic (e.g., ephedrine), anticancer (e.g., vincristine), antihypertensive (e.g., reserpine), antipyretic (e.g., quinine), and antihyperglycemic (e.g., piperine) effects [5].

Plant-derived alkaloids have been discovered to show anti-inflammatory activities by suppressing a range of pro-inflammatory protein complexes implicated in inflammatory signaling pathways. This complex includes nuclear factor-kappa-light-chain-enhancer of activated B cells (NF-kB), extracellular signal-regulated protein kinase 1/2 (ERK1/2), Akt, and signal transducer and activator of transcription 1(STAT1) as well as inflammatory mediators, that is, prostaglandin E2 (PEG2), nitric oxide (NO), cytokines, and chemokines [6, 7]. Inflammatory condition is distinguished by immune cell infiltration, activation, and production of several inflammatory mediators and cytokines, in excessive amounts [8]. Overproduction of inflammatory mediators leads to various inflammatory disorders such as bowel disease, rheumatoid arthritis, and cardiovascular diseases, and associated diseases such as diabetes, cancer, chronic kidney disease, neurodegenerative disorders (NDDs), and aging [9]. Likewise, NDDs were the second leading cause of high mortality worldwide in 2016 [10]. Alzheimer's diseases, dementia, Parkinson's disease, and amyotrophic lateral sclerosis are frequently observed NDDs. By 2040, they are predicted to overtake cancer as the second greatest cause of death following cardiovascular disease, according to the World Health Organization [11]. Alkaloids have been shown to improve the pathophysiology of NDDS by acting as monoamine oxidase (MAO) inhibitors, acetylcholinesterase, and butyrylcholinesterase inhibitors, and *N-methyl-D-aspartate* (NMDA) antagonists as well as muscarinic and adenosine receptor agonists [12].

Many pieces of evidence show that traditional medicine formulations, mostly made from plant-based components, could help treat inflammation, allergic disorders, and NDDs with minimum systemic toxicity. Therefore, this review highlights the specific role of alkaloids to modulate inflammatory and neurological conditions and aid the constant search for alkaloid-based therapy of both inflammatory and neurodegenerative diseases.

## 2. Methodology

**Figure 1:**
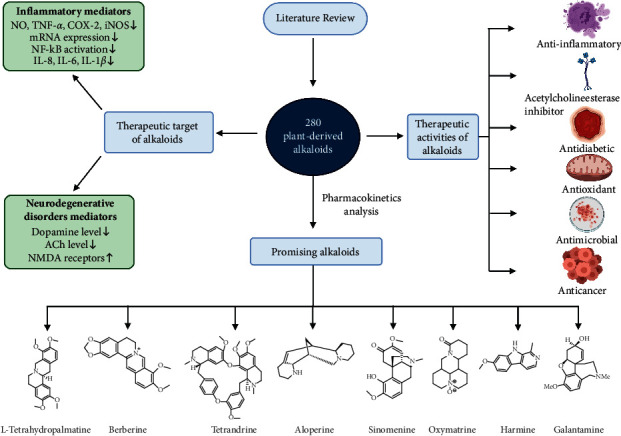
Schematic workflow of the study (note: TNF-*α* = tumor necrosis factor-alpha, IL-1*β* = interleukin-1 beta, IL-8 = interleukin-8, IL-6 = interleukin-6, COX-2 = cyclooxygenase-2, iNOS = inducible nitric oxide synthase, NO = nitric oxide, ACh = acetylcholine, NMDA = *N*-methyl-*D*-aspartate).

We have collected scientific information about inflammation, NDDs, and other health complications from peer-reviewed articles published in scientific journals. Mostly, medicinal plants and phytochemicals were considered during the study. To obtain the relevant data, Google Scholar, PubMed, Scopus, Science-Direct, conference papers, AMED, Cochrane Library, and other electronic literature databases were searched using the terms “alkaloids,” “medicinal uses,” “anti-inflammatory,” “analgesic,” “phytochemicals,” “medicinal plants,” “natural products,” “herbal,” “ethnopharmacology,” “neurodegenerative disorders,” etc. All the results were tallied via the utmost range of accessible literature. The potential pharmacokinetic properties such as absorption, distribution, metabolism, excretion, and toxicity (ADMET) of 280 alkaloids obtained during the literature survey were evaluated through the chemoinformatic tool pkCSM [13]. Furthermore, computational-based toxicity of the alkaloids was examined through the ProTox-II webserver, which gives information on various levels of indicators such as organ toxicity, oral toxicity, toxicological endpoints (carcinogenicity, cytotoxicity, hepatotoxicity, immunotoxicity, and mutagenicity), toxicological pathways, and active targets with a confidence score [14].  Figure 1 shows the schematic progress of the study.

## 3. Biosynthesis and Physiological Role of Plant-Derived Alkaloids

**Figure 2:**
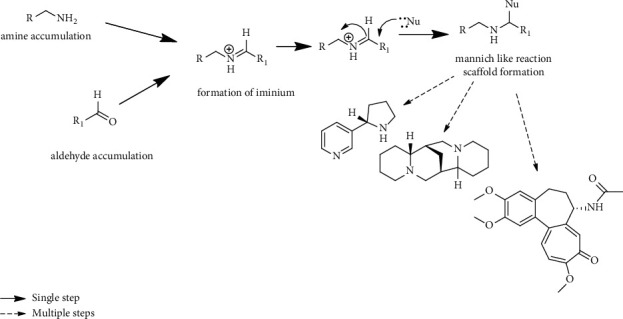
Basic biosynthetic route of plant-derived alkaloids.

The biosynthesis route of plant-derived alkaloids involves different synergistic steps, differentiated based on whether they are intramolecular or intermolecular. Its biosynthesis usually starts with amino acid precursors such as ornithine, arginine, lysine, phenylalanine, tyrosine, and tryptophan [15] or aldehyde precursors, followed by the generation of an iminium cation and then a Mannich-like reaction. This final step is sometimes referred to as the scaffolding step or the first dedicated step in a pathway as shown in Figure 2 [16]. The precursors from which alkaloids are biosynthesized are shown in Table S1.

Pyrrolizidine alkaloids, which are esters between necine bases derived from arginine or ornithine using putrescine intermediates and tiglic acid, and related C5 necine acids are derived from isoleucine in which homospermidine synthase catalyzes the initial step in biosynthesis. The majority of such alkaloids have been exclusively observed in *Senecio* species [17]. Biosynthesis of quinolizidine occurs within the genus *Lupinus* and some species of legumes, including *Lupinus angustifolius, L. luteus, L. albus,* and *L. mutabilis* [18]. Quinolizidine alkaloids are biosynthesized with the decarboxylation of lysine by lysine decarboxylase [19] to produce cadaverine amine, which on oxidation with amine oxidase generate spontaneous intramolecular Schiff base formation, resulting in a ring formation, and the addition of various functional groups yields the final product [18]. Furthermore, Lotus (*Nelumbo nucifera*) predominantly accumulates benzylisoquinoline [20], and biosynthesis of the majority of these alkaloids is biologically synthesized by the decarboxylation of tyrosine or L-dihydroxyphenylalanine (L-DOPA) to yield 4-hydroxyphenylacetaldehyde (4-HPAA) and L-dopamine, respectively, catalyzed by tyrosine/DOPA decarboxylase, followed by the condensation of L-dopamine and 4-HPAA to form (*S*)-norcoclaurine [21]. Similarly, the biosynthesis of ophiorrhines A and B, and monoterpenoid alkaloids was found via (4 + 2) Diels–Alder cycloaddition reaction forming two different intermediates. The first intermediate involves the oxidation of 5-oxodolichantoside, whereas the second intermediate, that is, 3-keto intermediate, is formed by the condensation of tryptamine and secologanin, and then, imine ion is formed from N-methylation and oxidation [22]. Pyruvic acid reductase plays an important role in tropane alkaloid synthesis. In this process, tropinone formed from putrescine is reduced to tropine, which on condensation with phenyl lactic acid gives littorine. Phenyl pyruvic acid reductase (PPAR) reduces phenyl pyruvic acid formed from littorine into phenyl lactic acid; thus, formed phenyl lactic acid is converted into hyoscyamine aldehyde, the precursor of anisodamine and scopolamine [23].

The initial step in the biosynthesis of benzylisoquinolines is the conversion of L-tyrosine to dopamine and 4-hydroxyphenylacetaldehyde, which is catalyzed by P450, and 6-*0*-methyltransferase, *N*-methyl transferase, and 4′-*O*-methyltransferase catalyze the methylation process to form the intermediate (*S*)-reticuline, it produces different benzylisoquinoline alkaloid (BIA) on multistep transformation [24]. The holy lotus genome has been shown to contain genes for BIA synthesis, norcoclaurine synthase (NCS), *O*- and *N*-methyltransferases, and cytochrome (CYP) monooxygenases CYP80A, CYP80G, and CYP719A [25], while the enzyme CYP80G associated with aporphine alkaloid biosynthesis, which catalyzes the conversion of (*S*)-reticuline to (*S*)-corytuberine through intramolecular C-C coupling [26]. Enzyme alcohol dehydrogenase (ADH) and cytochrome P450 (CYP450) catalyzed the conversion of strictosidine aglycone into the strychnos alkaloids akuammicine and acted as an important precursor for anticancer agent vinblastine and vincristine [27]. Similarly, the 4-(1-methyl-2-pyrrolidinyl)-3-oxobutanoic acid acts as an intermediary in tropinone formation, showing that the tropinone is synthesized by a polyketide synthase after two rounds of decarboxylation by malonyl-CoA [28]. Alkaloids have been proved to be the most effective against protective agents of metabolic operations and often act as neurotransmitters and signaling systems. They have been used to treat various disorders, including inflammation, allergies, cancer, diabetes, and many others [29]. Along with these, alkaloids play a crucial role in protecting from extreme temperature, salinity, water, radiation, heavy metals, and herbicidal injury [30].

## 4. Therapeutic Targets of Alkaloids

### 4.1. Inflammatory Mediators

Inflammation is a nonspecific and immediate response mechanism of the body's innate system against infectious (bacteria, viruses, fungi, and parasites) and noninfectious stimuli. Innate immune cells include white blood cells such as dendritic cells, neutrophils, natural killer cells, monocytes/macrophages, eosinophils, and basophils. Innate immune cells can produce and release pro-inflammatory mediators such as NO, cytokines, chemokines, hormones, growth factors, and adhesion molecules to sustain their communication and orchestrate immune responses [31].

Nuclear factors, such as NF-*κ*B, a family of inducible transcription factors, play a significant role in the inflammatory process because they regulate genes involved in immunological and inflammatory responses [32]. NF-*κ*B is responsible for the transcriptional induction of several cytokines, chemokines, growth factors, cell adhesion molecules, and some acute-phase proteins. The activation of NF-*κ*B involves the phosphorylation and degradation of its inhibitory protein via proteasomal degradation. As a result, free NF-*κ*B is released into the nucleus, where it binds to *κ*B binding sites in the promoter site of target genes, causing pro-inflammatory cytokine transcription. The major cytokines it regulates include tumor necrosis factor (TNF)-*α*, interleukin (IL)-1, IL-6, IL-8, and IL-12 Il-18. Furthermore, it also regulates the expression of chemokines, such as monocyte chemoattractant protein-1 (MCP-1), macrophage inflammatory protein-2 (MIP-2), C-X-C motif chemokine ligand (CXCL)-1, and CXCL-10 [33]. NF-*κ*B also modulates the inflammasome, a multiprotein complex composed of pattern recognition receptors that act as an innate immune system sensor to infectious microorganisms and host inflammatory proteins [34].

Overall, positive feedback mechanism and inflammatory cytokines again activate NF-*κ*B in innate immune cells, thereby inducing cytokines and chemokines in greater quantity leading to further infiltration of inflammatory cells to disseminate the inflammation [35]. This reaction further helps on-site differentiation and infiltration of adaptive immune cells to eliminate microbes and harmful antigens [36]. Usually, inflammation and release of inflammatory mediators are beneficial to the host as it helps resolve the diseases; however, the dysregulation of the inflammatory responses results in severe tissue damages and contributes to the development of acute or chronic inflammatory diseases. Therefore, in the later case, the inhibition of inflammatory mediators or their receptors remains beneficial to prevent further tissue damage.

**Figure 3:**
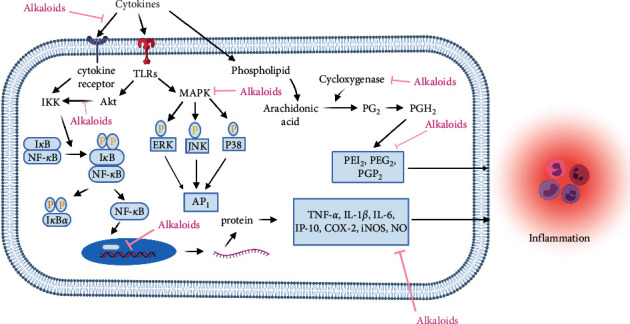
Mechanisms of plant-derived alkaloids in the treatment of inflammation (promotion ↑, inhibition ⊥). (Note: TLRs = toll-like receptors, IKK = IkB kinase, NF-kB = nuclear factor-kappa B MAPK = mitogen-activated protein kinase, ERK = extracellular signal-regulated kinase, JNK = c-Jun N-terminal kinase, AP-1 = activator protein-1, PGH2 = prostaglandin H2, PEG2 = prostaglandin E2, TNF-*α*: tumor necrosis factor-alpha, IL-1*β* = interleukin-1 beta, COX-2 = cyclooxygenase-2, iNOS = inducible nitric oxide synthase, NO = nitric oxide).

Prostaglandins are also important in inducing an inflammatory response. They are the lipids biosynthesized in inflamed and damaged tissue and, therefore, associated with the development of acute inflammation symptoms, swelling, and redness. The generation of prostaglandins from arachidonic acid, an essential fatty acid, involved cyclooxygenase (COX) isoenzymes. The prostaglandin synthesis is blocked by nonsteroidal anti-inflammatory drugs (NSAIDs), inhibiting COX activities [37]. Nitric oxide (NO) activates and regulates COX enzyme activity during the inflammatory condition [38], besides targeting prostaglandins. Hence, NO and/or COX are also common possible therapeutic targets to suppress the inflammatory pathogenesis of diseases.  Figure 3 illustrates the mechanism of alkaloids produced from plants in treating inflammation.

### 4.2. Mediators of Neurodegenerative Disorders (NDDs)

The continuous and irreparable damage of the structure or function of neurons coupled with pathologically changed proteins that accumulate in the human brain, leading to neuron degeneration, is known as neuron degeneration disease (NDD) [39]. The neuronal cell death may lead to different NDDs, such as Alzheimer's disease, Parkinson's disease, amyotrophic lateral sclerosis, Huntington's disease, brain trauma, progressive supranuclear palsy, prion diseases, and spinocerebellar ataxias. The common mechanism underlying these NDDs includes proteasomal dysfunction, as a result of which misfolded protein cleared insufficiently and correctly, leading to its aggregation in the brain. Furthermore, oxidative stress and production of free radicals/reactive oxygen species (ROS), mitochondrial malfunction and DNA repairs, fragmentation of neuronal cellular complexity, and neuroinflammation are directly associated with NDD etiology [40].

**Figure 4:**
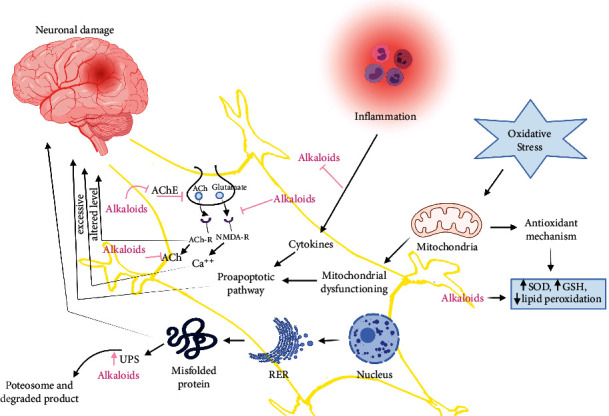
Mechanisms of plant-derived alkaloids in treating neurodegenerative disorders (increase ↑, inhibition ⊥). (Note: AChE = acetylcholinesterase, ACh = acetylcholine, ACh-R = acetylcholine receptor, NMDA-R= *N*-Methyl-*D*-Aspartate receptor, UPS = ubiquitin-proteasome system, SOD = superoxide dismutases, GSH = glutathione, RER = rough endoplasmic reticulum).

The presence of acetylcholinesterase (AChE) is linked to a plaque of extracellular *β*-amyloid protein (A*β*) deposits and neurofibrillary tangles in Alzheimer's disease, the most common NDD. A*β* is a small polypeptide generated from the processing of a larger transmembrane *β*-amyloid precursor protein (APP) by *β*-site amyloid precursor protein cleaving enzyme (BACE-1) [41]. To treat Alzheimer's disease, administered drugs can only reduce the symptoms or delay disease progression by inhibiting AChE [42]. Alkaloids could function as a neuroprotective agent by inhibiting several cellular activities, such as inhibiting AChE enzyme activity, increasing the level of gamma-aminobutyric acid (GABA), an inhibitory neurotransmitter in the mammalian brain, by partially blocking NMDA receptors and enhancing cellular autophagy function and many more mechanisms [12]. Similarly, the etiology of Parkinson's disease, another common NDDs, involves genetic, nongenetic [43], and environmental factors [43]. The common characteristics are an accumulation of misfolded protein aggregates, proteasomal dysfunction, mitochondrial DNA damage, oxidative stress, neuroinflammation, and genetic mutations. The hallmark of Parkinson's disease is the dopaminergic neuronal loss in the substantia nigra pars compacta (SNpc) and reduced dopamine levels [44]. Several investigations have found that numerous misfolded protein aggregates, such as A*β*, p-tau, and *α*-synuclein, are frequently observed in human post-mortem brains of patients with mixed dementia with Lewy bodies and Parkinson's disorder with dementia [45]. Catalyzing dopamine by the enzyme monoamine oxidase-B (MAO-B), which is increased in the brain, is the cause of decreased dopamine levels, hence indicating MAO-B is a good target to maintain dopamine levels in Parkinson's diseases [46].  Figure 4 explains the mechanism of the plant-isolated alkaloids for medicating neurodegeneration.

## 5. Ethnopharmacological Survey of Plants Producing Therapeutic Alkaloids

**Figure 5:**
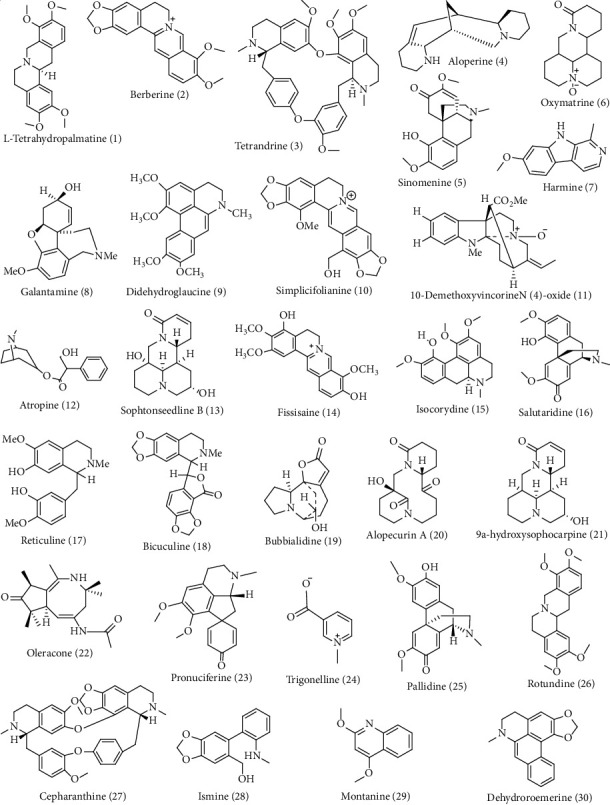
Selected plant-derived alkaloids isolated from various medicinal plants.

Many modern pharmaceutical treatments are derived from traditional and natural remedies. Humans have relied on herbs and plants as sources of effective medicine for treating illness for thousands of years. In conventional medicine, more than 53,000 plant species from worldwide have been used [47]. For instance, *Berberis vulgaris*, rich in berberine alkaloids, is traditionally used as a herb that is effective in preventing coronary artery disease and shows anti-inflammatory and immunomodulatory effects. The trigonelline alkaloid, isolated from *Trigonella foenum-graecum* in China, is effective for curing diabetes and central nervous system (CNS) diseases [48]. Due to synergistic actions of secondary ingredients, such as oxindole and L-stachydrine from *Capparis tomentosa* has been traditionally used for inflammation in Tanzania [49]. Similarly, the traditional Chinese herbal plant *Lycoris radiata*, natural isoquinoline alkaloid, has different biological utilities, including anti-inflammatory-related activity [50]. Similarly, evolitrine, a key component identified in the leaves of *Acronychia pedunculata*, a traditional medicinal plant in Sri Lanka, was discovered to have NO inhibitory, anti-nociceptive, antihistamine, and antioxidant properties [51]. Anti-inflammatory and antioxidant activities have been revealed in alkaloids found in Malaysian and Thai *Erythroxylum cuneatum l*eaf extract [52]. *Neolamarckia cadamba*, a plant traditionally used in China to treat inflammation, fever, and itch, contained 3-dihydrocadambine, a key chemical with anti-inflammatory efficacy *in vitro* and *in vivo* studies [53]. The acridone alkaloids obtained from the barks of *Citrus aurantium* have been reportedly used in Nigerian traditional medicine to treat inflammatory diseases and for the management of cancer [54]. Similarly, mistletoe, i.e., *Viscum album L.,* a culturally significant plant in Europe, has been used to treat neurological conditions such as epilepsy, hysteria, nervousness, and Alzheimer's diseases [55]. Likewise, the aerial part of the plant *Sida acuta* (Malvaceae family), used in traditional Ayurvedic Indian medicine, contained a good quantity of cryptolepine, which has anti-inflammatory properties [56]. In addition, *Stephania rotunda* (Menispermaceae) has been utilized as a folk medicine in several Asian nations, which is prevalent in hilly regions of Cambodia and found to contain several bisbenzylisoquinoline alkaloids like 2-norcepharanthine, cepharanoline, and fangchinoline that is used for the management of inflammatory diseases [57]. Around 392 species of African plants have been found to possess isoquinoline alkaloids (19%) and have been used for the exclusive treatment of cancer and inflammation-related diseases [58]. Sceletium contains alkaloids of the mesembrine type, including Δ^7^ mesembrenone, mesembranol, mesembrenone, mesembrine, and epimesembranol, which are used as a traditional medicine in South Africa to treat neurodegenerative disease [59]. In the Guangxi and Yunnan provinces of China, *Stephania cepharantha* has been widely used to treat stomach aches and snakebites, and the alkaloids present in it have been found to possess potential anti-neuroinflammatory agents [60]. We have also tried to report and shed light on the listed alkaloids in Table S2 comprehensively. Furthermore, the structures of the topmost 30 alkaloids based on ADMET and ProTox-II are shown in Figure 5. The structure of the rest of the alkaloids taken in this review is shown in Figure S1.

Overall, ethnopharmacological surveys have contributed to identifying potential metabolites in traditionally used medicinal plants. Many of these metabolites have been studied for their pharmacological applications, leading to the invention of drugs and the use of metabolites in modern medicine.

## 6. Therapeutic Activities of Plant-Derived Alkaloids

**Figure 6:**
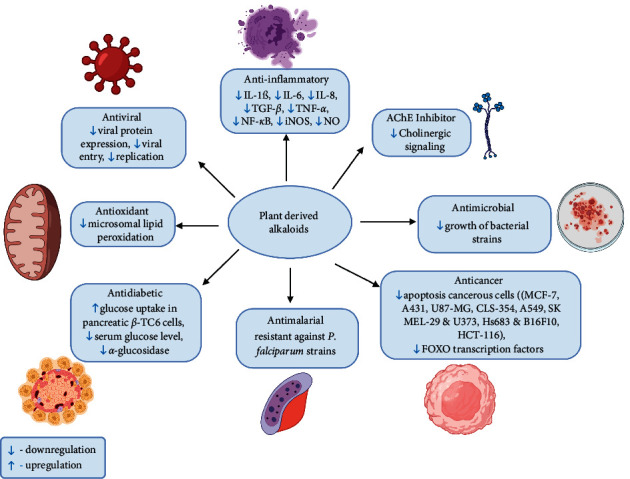
Biological applications of alkaloids (note: IL-1*β* = interleukin-1*β*, IL-6 = interleukin-6, IL-8 = interleukin-8, TGF-*β* = transforming growth factor-*β*, TNF-*α* = tumor necrosis factor *α*, NF-*κ*B = nuclear factor-kappa B iNOS = inducible nitric oxide synthase, NO = nitric oxide, MCF-7 = Michigan cancer foundation-7, U87-MG = uppsala-87 malignant glioma).

**Figure 7:**
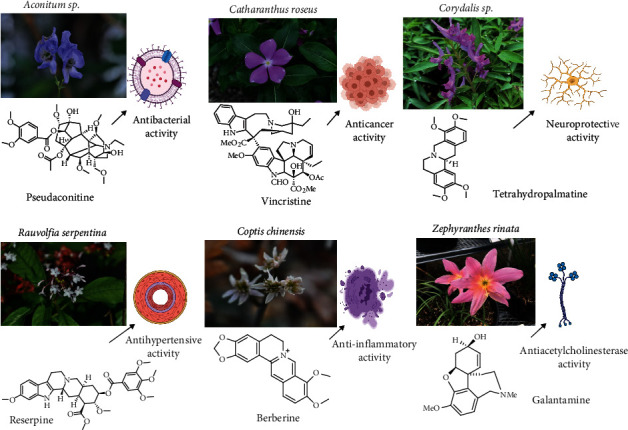
Diagrammatic graph shows some plants and their pharmacological applications.

In both traditional and modern medical systems, the biological activities of alkaloids such as anticancer, antibacterial, anti-inflammatory, antimicrobial, antioxidant, AChE inhibitory activity, antimalarial, and antidiabetic activity have been examined as shown in Figure 6. Likewise, Figure 7 shows the diagrammatic graph of some plants and their pharmacological applications.

Most plant-derived alkaloids have been demonstrated for antiproliferation, antiviral, antibacterial, insecticidal, and antimetastatic effects [61]. Due to the presence of protons receiving N-atoms and one or more protons donating amine H-atoms, alkaloids are also searched for their tendency to form hydrogen bonds with enzymes, receptors, and proteins in them [62]. Plant-derived alkaloids that show some biological activities are mentioned below.

### 6.1. Anti-Inflammatory Activity

Canthin-6-one alkaloids obtained from *Ailanthus altissima* stem bark have an anti-inflammatory effect by suppressing both NF-*κ*B transcriptional activations and the Akt phosphorylation [63]. The plant-isolated alkaloids, berberine, showed promising results for treating Acne Vulgaris as it decreased pro-inflammatory cytokines, that is, IL-1 *β*, IL-6, IL-8, and TNF-*α* [64]. The anti-inflammatory response of the compound can be retrieved based on the NO inhibitory impacts on lipopolysaccharide (LPS) stimulated macrophages model [65]. Out of 23 compounds isolated from the roots of *Isatis tinctoria,* the NO production analysis revealed that tryptanthrin, 3-(2-carboxyphenyl)-4(3H)-quinazolinone, and 2-methyl-4(3H)-quinazolinone displayed inhibitory effects with the respective half-maximal inhibitory concentration (IC_50_) values of 1.2, 5.0, and 74.4 *μ*M [66]. Likewise, ethanol extract of 1-carbomethoxy-*β*-carboline alkaloids from *Portulaca oleracea* showed the most effective anti-inflammatory activity [67] due to the stifling mitogen-activated protein kinase (MAPK) pathways and NF-*κ*B, lowering the production of pro-inflammatory mediators such as inducible nitric oxide synthase (iNOS), TNF-*α*, IL-6, and IL-1*β* [67]. The alkaloids extracted from Chinese medical herbs are applicable in treating rheumatic immune diseases by invigorating the discharge of adrenal cortex hormones, obstructing the unleash of cytokines, and synchronizing the degree of NO [68, 69]. Oleracimine showed attenuating effect by limiting the production of NO and reducing the expression of IL-6, TNF-*α*, and PEG2 in both messenger ribonucleic acids (mRNAs) the protein level by inhibiting the activity of COX-2 and NO synthase enzyme [70]. Hence, the numerous plant-based alkaloids have mounting effects in the treatment of anti-inflammatory activity.

### 6.2. AChE Inhibitory Activity

AChE was taken as a valuable spot for controlling NDDs causing cholinergic signaling deficit [71]. The alkaloids were extracted from the root of *Zanthoxylum rigidum*; nitidine and avicine showed inhibitory action against AChE with IC_50_ of 0.65 ± 0.09 *μ*M and 0.15 ± 0.01 *μ*M, respectively [72]. Another study showed four pyrrolizidine alkaloids, namely, 7-O-angeloylechinatine-N-oxide, 3′-O-acetylheliospine-N-oxide, heliosupine N-oxide, and heliosupine, isolated from *Solenanthus lanatus,* which possess the AChE inhibitory activity with IC_50_ values ranging from 0.0001–0.60 mM [73]. Among monoterpene indole alkaloids extracted from the leaves of *Rauvolfia vomitoria*, rauvomitorine III alkaloids showed anti-AChE activity with an IC_50_ value of 16.39 ± 1.41 *μ*M due to the existence of the *N*-methyl group in the vobasenal-type alkaloids as well as due to interactions with Trp133 and Trp86 moieties at hydrophobic appendages [74]. Similarly, mokluangin A-C and antidysentericin alkaloids extracted from the bark of *Holarrhena pubescens* showed strong AChE inhibitory activity with IC_50_ values ranging from 1.44 to 23.22 *μ*M [75]. The alkaloids such as galantamine, caranine, *N-*demethylgalanthamine, and lycoramine obtained from leaves, roots, and bulbs of Amaryllidaceae species, *Crinum, Habranthus, and Zephyranthes* act as the most significant AChE inhibitors in correlation with chemical fingerprints [76]. Galantamine was found the most promising dual-site binding AChE inhibitor via the combinatorial library of Galantamine-curcumin hybrids [77]. These findings indicate that the plant-derived alkaloids have potent anti-AChE inhibitory activity.

### 6.3. Antioxidant Activity

Antioxidants are substances that fight against free radicals in the cells, which otherwise highly contribute to developing heart disease, cancer, and other diseases [78–80]. The phytochemical investigation of *Nelumbo nucifera* embryos revealed the antioxidant activity of its four main alkaloids, named neferine, isoliensinine, liensinine, and armepavine. Using the 2,2′-azino-bis(3-ethylbenzothiazoline-6-sulfonic acid (ABTS) and 2,2,-diphenyl-1-picrylhydrazyl (DPPH) radical scavenging assays, the drug concentration eliciting 50% of the maximum simulation (SC_50_) values for these compounds was identified as 14.65, 12.07, 18.25, and 29.03 *μ*M for ABTS and 33.37, 25.26, 44.21, and 79.34 *μ*M for DPPH, respectively [81]. Similarly, among seven alkaloids isolated from *Alphonsea cylindrical* barks, iraqiine, muniranine, and kinabaline exhibit potent antioxidant activity against DPPH radical scavenging assay with half-maximal inhibitory concentration (IC_50_) values of 48.77 ± 1.01, 44.51 ± 1.12, and 64.28 ± 0.93 *μ*g/ml, respectively [82]. Similarly, from the root of *Stephania tetrandra,* 15 different alkaloids have been isolated, among which three new aporphine alkaloids and two new phenanthrene alkaloids were reported. These alkaloids, (+)-dicentrine, (+)-neolitsine, (+)-glaucine, (-)-nuciferine, and stephenanthrine, also exhibit antioxidant activities, which were measured by determining malondialdehyde levels in rat liver microsomal lipid peroxidation induced by Fe^2+^/cysteine with the inhibitory values ranging from 62.50 ± 1.91 to 98.44 ± 0.34% at the concentration of 10 *μ*M [83]. Likewise, alkaloids, mitraphylline, and isomitrphylline isolated from aqueous leaf extract of *Uncaria tomentosa* showed antioxidant activities, evaluated through DPPH, ABTS, & ferric ion reducing antioxidant power (FRAP) assay [84]. These overall findings indicate that plant-derived alkaloids possess antioxidant activity.

## 7. ADMET and ProTox-II Analysis

*In silico* ADMET studies are the most important considerations in drug discovery and development concerning pharmacokinetics properties [85]. To evaluate the alkaloids as promising therapeutic, pharmacokinetic properties ADMET (Table S3) and ProTox-II (Table S4) are analyzed. On predictive pkCSM, intestinal absorption values greater than 30% are considered to be better absorbed from the intestine after oral administration, which indicates that all alkaloids were able to be remarkably absorbed from the intestine of humans. Likewise, compounds with logPapp >0.90 are considered with high CaCO_2_ permeability. Compounds with log blood-brain barrier (logBBB) < −1 are weakly dispersed to the brain, whereas those with logBBB > 0.3 can pass the BBB. As indicated, compounds **2, 4, 7, 8, 12, 30,** etc. were able to readily cross the BBB, whereas other compounds were unable to do so. Another important parameter, the volume of distributions (VD), is considered when logVDss < −0.15 and high when logVDss > 0.45 [13, 86]. Thus, the computational analysis showed relatively low water solubility, moderate CaCO_2_ permeability, and high intestinal absorption value for the following alkaloids: tetrahydropalmatine (**1**), berberine (**2**), tetrandrine **(3**), aloperine (**4**), sinomenine (**5**), oxymatrine (**6**), harmine (**7**), and galantamine (**8**). Furthermore, CYP (1A2, 2C9, 2C19, 2D6, and 3A4) parameters analyzed by ADMET are related to phase-1 drug bioinformatics in the metabolism of the drug [87]. The most important aspect of this study is CYP3A4, in which the alkaloids such as oxymatrine (**6**), bicucine (**18**), and pallidine (**25**) only inhibited it, whereas none of the other alkaloids were able to inhibit CYP3A4. This indicates that these mentioned alkaloids can be metabolized in the liver. It has an impact on total clearance and half-life. The total clearance describes the association between drug clearance rate and drug concentration in the body [88]. Compounds **1**, **2**, **10**, **12**, **14**, **16-17**, **19**, **20**, **25-26**, etc. were found to show high clearance. Additionally, compounds with log PS > −2 would penetrate the central nervous system (CNS) and act as CNS-active drugs. Moreover, AMES toxicity is also an important parameter in selecting the drugs. Compounds **4, 5, 7, 27**, **37, 38, 42, 45, 46, 49, 50, 66-70, 73, 75, 78-85, 87-89, 96-99, 104, 112, 113, 116, 118, 125, 128, 130-132, 134-136, 139, 141, 172, 174, 176, 179, 185-188, 195, 197, 204, 205, 212, 213, 218, 221, 223-229, 236, 240, 271, 273, 274,** and **275** were found with AMES toxicity. The toxicity of secondary metabolites was evaluated using ProTox-II based on toxicity and lethal dose (LD_50_) values ranging from class 1 and 2 (fatal), class 3 (toxic), class 4 and 5 (harmful), and class 6 (non-toxic) [14]. The ADMET and ProTox-II properties of tetrahydropalmatine, berberine, tetrandrine, aloperine, sinomenine, oxymatrine, harmine, and galantamine were optimal within the categorical range in comparison with nicotine (Alzheimer's disease: NCT00018278 and Parkinson's disease: NCT01216904) [89].

## 8. Promising Plant-Derived Alkaloids

### 8.1. Tetrahydropalmatine

**Figure 8:**
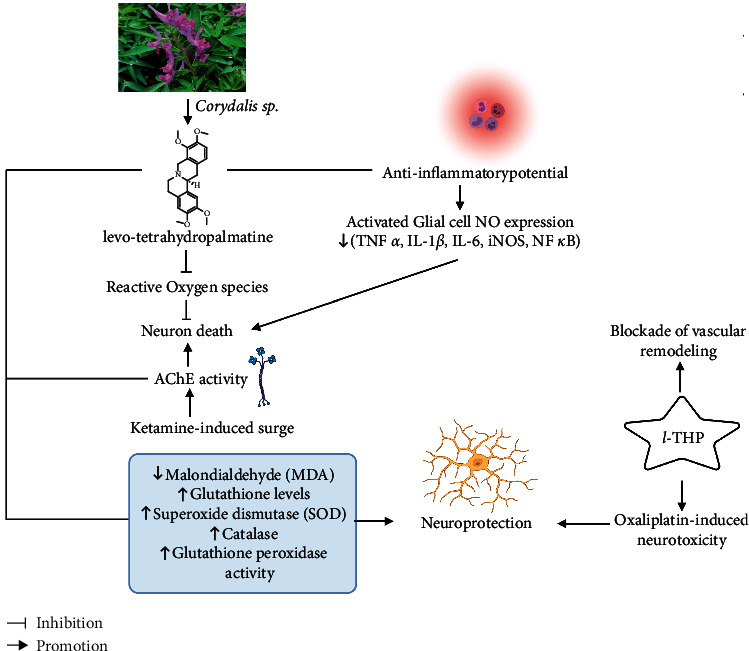
Anti-inflammatory and neurodegenerative mechanism involved in Levo-tetrahydropalmatine (*l*-THP). (Note: TNF-*α* = tumor necrosis factor *α*, IL-1*β* = interleukin-1*β*, IL-6 = interleukin-6, iNOS = inducible nitric oxide synthase, NF-*κ*B = nuclear factor-kappa B).

Tetrahydropalmatine (THP), an isoquinoline alkaloid, mainly extracted from *Stephania* and *Corydalis* genus, depicts anxiolytic, anti-inflammatory, analgesic, and cardioprotective activities [90, 91]. THP attenuated ketamine-induced surge in AChE activity, thus overruling the ketamine-induced decrease in ACh levels, demonstrating the protective action against nerve cell apoptosis in ketamine-induced mice [92]. Studies revealed that THP treatment rectified D-galactose-induced memory impairments associated with the decrease in malondialdehyde (MDA) and NO levels and increase in glutathione levels, and superoxide dismutase (SOD), catalase, and glutathione peroxidase activities [93]. Similarly, THP treatment showed a protective effect against ketamine-induced oxidative stress in mice, increasing glutathione peroxidase and SOD activities and decreasing MDA activities. Moreover, THP lowered TNF-*α*, IL-1*β*, and IL-6 expression-suppressed iNOS and NF-*κ*B protein activities and induced glial cell-derived neurotrophic factor protein expression in ketamine-induced mice, demonstrating its anti-inflammatory actions. *l*-THP blocked TNF-*α*-induced adhesion of monocytes to human umbilical vein endothelial cells by inhibiting the production of both mRNA and protein levels of vascular cell adhesion molecule-1 (VCAM-1) along with the attenuation of TNF-*α*-stimulated NF-*κ*B translocation in monocytes, highlighting its potential pharmacological action to intervene atherosclerosis [94].  Figure 8 shows the anti-inflammatory and neurodegenerative mechanism of *l*-THP. Thus, extensive pharmacological studies should be carried out further for rationalizing its anti-inflammatory and neuroprotective actions.

### 8.2. Berberine

Berberine shows anti-inflammatory activity by reducing the pro-inflammatory response via the activation of AMP-activated protein kinase (AMPK) in macrophages and suppressing the expression of pro-inflammatory genes such as TNF-*α*, IL-I*β*, IL-6, monocyte chemoattractant protein-1 (MCP-1), COX-2, and iNOS [95]. Most importantly, berberine also inhibits the production of TNF-*α* and IL-6 in HepG2 cells, illustrating its anti-inflammatory activity in hepatocytes [96].

The use of berberine has been widely studied in the NDDs model. In a rat model of Alzheimer's disease, berberine chloride prevented neurodegeneration of the hippocampus and decreased the activity of BACE-1 [97]. In the transgenic mouse model of Alzheimer's disease, berberine significantly reduced A*β* plaque aggregation leading to the improvement of neuronal and mental disturbance by the inhibition of APP phosphorylation [98]. Moreover, oral administration of 50 mg/kg berberine for 5 weeks in Parkinson's disease mice managed memory loss symptoms by reducing apoptosis in the hippocampus and prohibiting dopaminergic neuronal loss in substantia nigra [99]. Currently, berberine is at different clinical trial phases to treat various diseases, including atherosclerosis (NCT03470376), hypercholesterolemia (NCT02078167), schizophrenia (NCT03470376), coronary artery disease (NCT03378934), and Alzheimer's disease (NCT03221894) [89]. Thus, berberine must be a significant drug for treating inflammatory and neurodegenerative diseases.

### 8.3. Tetrandrine

Tetrandrine is a bisbenzylisoquinoline alkaloid isolated from the roots of *Stephania japonica*, *S. tetrandra,* and *S. Moore* with a wide usage in treating inflammation [100]. Tetrandrine has been identified to inhibit the secretion of pro-inflammatory mediators, TNF-*α*, IL-6, and IL-1*β* expression by blocking the NF-kB signaling in LPS-induced macrophages [101]. Also, tetrandrine inhibits the expression of tissue metalloproteinase inhibitor-1, matrix metalloproteinase-3, and the production of PEG2 (prostaglandin E2) and NO (nitrite oxide) by inhibiting IkB*α* phosphorylation in ATDC5 cells and LPS-induced cells [102]. Similarly, intragastric administration of tetrandrine decreased the concentration of NO in serum and pancreatic tissue of the acute hemorrhagic necrotizing pancreatitis rat model, and it inhibited the activation of NF-*κ*B by targeting the formation of IL-8, TNF-*α*, and IL-6 [103]. Likewise, the administration of tetrandrine proceeded intravenously in a rat model of Alzheimer's disease showed improvement in memorial and learning disability along with the decrease in the expression of TNF-*α* and IL-1*β* through the inhibition of the NF-*κ*B pathway [104]. Interestingly, recently, a phase 4 clinical trial of tetrandrine tablets or tetrandrine has been used to treat COVID-19 patients (NCT04308317) as an anti-inflammatory drug [89]. Overall, the above evidence demonstrates that tetrandrine is a potent alkaloid in treating inflammation and neurodegenerative disease.

### 8.4. Aloperine

Aloperine, a kind of piperidine alkaloid, has a therapeutic effect on inflammation and neuropathic pain. It is isolated from *Sophora alopecuroides*, *a* plant used as a medicine widely distributed in Central and Western Asia [105, 106]. The ability of aloperine to inhibit the Toll-like receptor 4 (TLR4)-dependent inflammatory pathway in macrophages demonstrated its anti-inflammatory activity. It was thus shown to block the expression of TNF-*α*, IL-17A, and IL-6 and reduce the secretion of PEG2 via COX-2 and iNOS inhibition, consequently lowering NO production [107]. Similarly, aloperine treatment also reduced oxidized low-density lipoprotein, a marker of endothelial inflammation, and reduced MCP-1, VCAM-1, IL-6, and E-selection by reducing Kruppel-like factor 2 (KLF2) expression, suggesting the potential anti-atherosclerosis characteristics [105]. Further evidence shows that 80 mg/kg of aloperine was injected intraperitoneally that decreased neuropathic pain possesses by chronic constriction injury in the dorsal spinal cord by inhibiting the upregulation of NF-*κ*B, IL-1*β*, and IL-6, which is related to the reduction of ROS through the suppression of NF-*κ*B pathways [106]. Even though aloperine has a wide range of medical applications, additional research is necessary to develop aloperine as a drug for treating various diseases, including inflammation and neurodegenerative diseases.

### 8.5. Sinomenine

Sinomenine is a kind of benzyl alkaloid commonly found in Chinese herbal medicine that is mainly isolated from the root and stem of *Sinomenium acutum* [108]. It has a therapeutic application in treating chronic nephritis, rheumatoid arthritis, myocardial ischemia, ankylosing spondylitis, and other rapid arrhythmias and reduces associated foot swelling caused by formaldehyde, egg white, or carrageenan [109]. The anti-inflammatory activity of sinomenine is through the inhibition of c-Jun N-terminal kinases (JNKs) and NF-*κ*B signaling pathways, thus suppressing the mRNA expression of cytokines, such as IL-1*β* and TNF- *α* [110]. *In vivo* study showed that 40 mg/kg of sinomenine administration on mice with experimentally induced rheumatoid arthritis remarkably reduced mechanical hypersensitivity during the peak of inflammation and the post-inflammatory phase [111].

Similarly, sinomenine also stands to have a good therapeutic value against NDDs. It has been shown to inhibit ROS and NO generation in A*β*-treated human astrocytes, implying a favorable effect in Alzheimer's disease [112]. It also accounted for significant neuroprotective potential in the rat model induced with temporal lobe epilepsy in intrahippocampal kainate by decreasing the intensity of seizures, the incidence of status epilepticus, hippocampus abnormal mossy fiber sprouting (MFS), and deoxyribonucleic acid (DNA) fragmentation [113]. Clinical studies showed that 101 out of 120 patients improved rheumatoid arthritis by combining sinomenine with methotrexate as a therapeutic agent [114]. Therefore, sinomenine is an effective alkaloid for treating both inflammation and neurodegenerative disease by reducing the expression of pro-inflammatory cytokines.

### 8.6. Oxymatrine

Oxymatrine is a quinolizidine alkaloid extracted from the roots of *Sophora flavescentis* that has anti-inflammatory, antiallergic, antiviral, antifibrotic, anticancer, and cardiovascular protective properties [115]. Since oxymatrine reduced the production of cytokines, TNF-*α*, and IL-17A, as well as lowered the arthritic score and synovial inflammation, it has been shown to have a potent anti-inflammatory effect on collagen-induced arthritis (CIA) rats [116]. Oxymatrine carries remarkable protective effects on gastric ulcers via the suppression of gastric inflammatory reactions, oxidative stress, and pro-apoptotic actions. It was found to prevent several inflammatory mediators in ulcered tissue by blocking NF-*κ*B translocation from the cytoplasm to the nucleus [117]. Oxymatrine is also reported for its anti-Alzheimer's disease effects by downregulating the densities of A*β* plaques and astrocyte clusters along with the improvement in the learning and cognitive abilities in the mice model [118]. Furthermore, by suppressing apoptosis and oxidative stress, it had effective neuroprotection against cerebral hypoxic-ischemic injury, which could be linked to the activation of protein kinase (Akt) and glycogen synthase kinase-3 (GSK3) and modification of the nuclear factor erythroid 2-related factor 2/heme oxygenase 1 (Nrf-2/HO-1) signaling pathway [119]. Through cathepsin-D-dependent regulation of the Toll-like receptor 4 (TLR4) signaling pathway, oxymatrine significantly attenuates 1-methyl-4-phenyl-1,2,3,6-tetrahydropyridine-induced Parkinson's diseases, and this provides dopamine-based neuroprotection and reduces microglia-mediated neuroinflammation [120]. It has been found as a potent anti-inflammatory and neurodegenerative drug, as 12 patients who have psoriasis (a type of skin inflammation) treated by the use of oxymatrine in the duration from 2012 to 2016 showed more significant improvement by inhibiting the excessive secretion of cell proliferation marker in skin surface [121]. With these findings of oxymatrine, further investigations with a wide range of therapeutical applications are in progress.

### 8.7. Harmine

Harmine, a *β*-carboline alkaloid, possesses considerable pharmacological importance such as anti-inflammatory, hallucinogenic, antioxidant, antitumor, antifungal, and antibacterial. The seeds of *Peganum harmala* were firstly used for its isolation [122]. The anti-inflammatory activity of harmine was demonstrated in LPS-injected mice. It was found to inhibit NF-*κ*B activation, which lowered the serum level of IL-1*β*, TNF-*α*, and IL-6 [123]. Harmine-loaded ethosomes are beneficial for treating inflammation in a rat paw edema induced by carrageenan, potentially inhibiting the expression of inflammatory factors such as PEG-2, TNF-*α*, IL-1*β*, and NO [124]. Furthermore, it also downregulated TLR4 and nucleotide-binding oligomerization domain (NOD), leucine-rich repeat (LRR), and pyrin domain-containing protein 3 (NLRP3) expression myeloperoxidase activity and MDA production along with the enhancement in the activities of SOD [125]. TNF-*α*, NO generation, and myeloperoxidase activity were all reduced, as a result of which scopolamine-induced inflammation was lowered [126]. It inhibited NLRP3 inflammasome activation by reducing NLRP3, apoptosis-associated speck-like protein containing a caspase-recruitment domain (ASC), cleaved caspase-1, IL-1*β*, and IL-18 levels, and thus, boosted the brain-derived neurotrophic factor/tropomyosin receptor kinase B (TrkB) signaling pathway, as a result of which cognitive impairment was attenuated in streptozotocin-induced diabetic rats [127]. Oral administration of 20 mg/kg harmine results in the improvement of memory impairment by increasing cholinergic function through the inhibition of AChE in scopolamine-induced mice, which shows that harmine may be potent for the treatment of neurodegenerative disease [128]. Drug harmine dimethyl-tryptamine (DMT) was used in clinical trials of 30 participants for the emotion mood cognitive function 1 (NCT04716335), and Social Empathy is in phase 4 clinical trial (NCT04716335) [89]. Therefore, harmine could stand as a potent anti-inflammatory and NDD drug.

### 8.8. Galantamine

**Figure 9:**
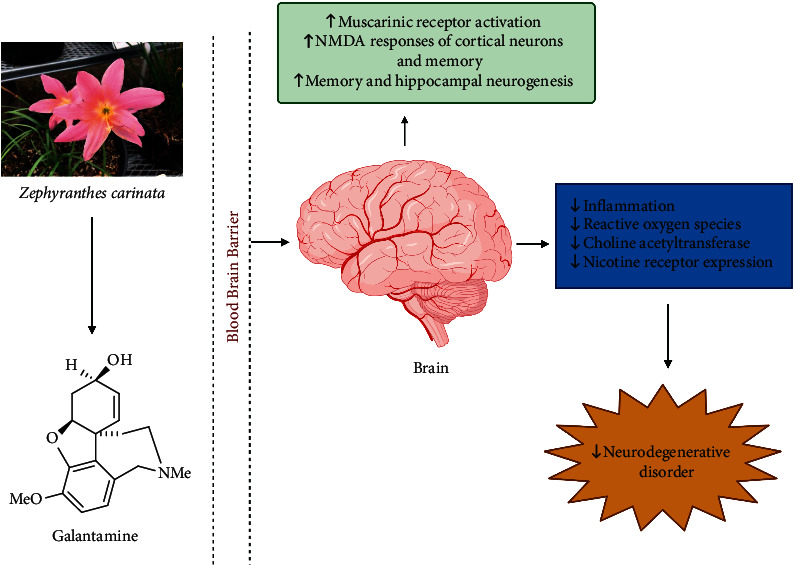
Involved mechanism of galantamine in treating the neurodegenerative disorder (promotion ↑, inhibition ⊥). (Note: NMDA = *N-*methyl-*D*-aspartate).

Galantamine is a plant alkaloid, commonly isolated from *Galanthus woronowii*, *Leucojum aestivum,* and other members of the family Amaryllidaceae [129]. It is approved by the US Food and Drug Administration as a medication for treating Alzheimer's disease and is available under brand names Reminyl and Nivalin [130]. The relevant mechanism of galantamine in treating neurodegenerative diseases is depicted in Figure 9. Inhibition of the gliosis, cytokines (IL6, IL-1*β*, and TNF-*α*), and pro-inflammatory signaling molecules (NF-*κ*B p65) and inflation of the synapse-associated proteins in the hippocampus of lipopolysaccharide-exposed mice mark galantamine as a promising treatment to ameliorate neuroinflammation and cognitive decline in neurodegenerative disorders [131]. Additionally, it displayed neuroprotective action through nicotinic receptors via the PI3K-Akt and Bcl signal transduction cascade [132]. Galantamine enhanced NMDA responses of rat cortical neurons, suggesting its importance in the improvement of learning/memory/cognition in Alzheimer's diseases patients [133]. It has been shown to induce hippocampal insulin-like growth factor 2 mRNA levels in mice on acute administration, suggesting its neurogenetic action [134]. Galantamine hydrobromide is in phase 3 clinical trials for Alzheimer, dementia, mental disorders, and brain diseases (NCT00216502) [89]. Thus, galantamine, which effectively inhibits the generation of cytokines and the expression of NF-*κ*B, can be utilized to treat Alzheimer's disease.

## 9. Conclusions

Alkaloids, one of the remarkable classes of natural compounds, exhibit extensive routes of structurally and/or functionally diverse molecules for the new potential preventative and/or therapeutical use in anti-inflammatory, AChE inhibition, and NDDs. Based on the literature survey and *in silico* ADMET analysis, alkaloids, namely, harmine, berberine, aloperine, oxymatrine, tetrandrine, sinomenine, tetrahydropalmatine, and galantamine, have the potential to serve as a lead compound against several anti-inflammation and NDDs. However, for further studies, it is imperative to explore extensively clinical trials, pharmacokinetic properties, health complications, and other important parameters before its medicinal applications. Many alkaloids are also toxic; thus, their safety profiles should be studied in detail. Collaborative research using alkaloids in combination with currently FDA-approved medicines could be investigated for improved and long-term anti-inflammatory and anti-AChE formulations. Hence, this review will be significant on medicinal chemistry, ethnopharmacological applications, and research on drug delivery regarding the alkaloids in the management of inflammation and NDDs.

## Data Availability

The data used to support the findings of this study are available from the corresponding author upon request.

## Abbreviations

AChE: Acetylcholinesterase
ADMET: Absorption, distribution, metabolism, excretion, and toxicity
A*β*: *β*-amyloid
Akt: Protein kinase B
ALS: Amyotrophic lateral sclerosis
AMPK: 5' adenosine monophosphate-activated protein kinase
CIA: Collagen-induced arthritis
COX: Cyclooxygenase
COX-2: Cyclooxygenase-2
CYP: O- and N-methyltransferases, and cytochrome
IL-1*β*: Interleukin-1*β*
IL-6: Interleukin-6
IL-8: Interleukin-8
JNKs: c-Jun N-terminal kinases
MAPK: Mitogen-activated protein kinase;
MCP-1: Monocyte chemoattractant protein-1
MDA: Malondialdehyde
NCS: Norcoclaurine synthase
NDDs: Neurodegenerative disorders
NF-*κ*B: Nuclear factor-kappa B
NMDA: N-Methyl-D-aspartate
PEG2: Prostaglandin E2
SOD: Superoxide dismutase
STAT-1: Signal transducer and activator of transcription 1
THP: Tetrahydropalmatine
TLR: Toll-like receptor
TNF-*α*: Tumor necrosis factor *α*
VCAM-1: Vascular cell adhesion molecule-1.

## Supplementary Materials

Supplementary Materials Table S1: plant-derived alkaloids with their biosynthetic precursors and current research. Table S2: occurrence, pharmacology, and toxicity of alkaloids. Table S3: ADMET properties of promising plant alkaloids by pkCSM server. Table S4: prediction of toxicity of secondary metabolites inhibiting metabolic enzymes using ProTox-II. Figure S1: molecule structure of plant-derived alkaloids.
