# Supplementary material for: Potential Therapeutic Applications of Plant-Derived Alkaloids against Inflammatory and Neurodegenerative Diseases
Source: Evid Based Complement Alternat Med. 2022 Mar 9;2022:7299778. doi: 10.1155/2022/7299778 (PMC8926539; doi:10.1155/2022/7299778)
Supplement: Supplementary Materials — Table S1: plant-derived alkaloids with their biosynthetic precursors and current research. Table S2: occurrence, pharmacology, and toxicity of alkaloids. Table S3: ADMET properties of promising plant alkaloids by pkCSM server. Table S4: prediction of toxicity of secondary metabolites inhibiting metabolic enzymes using ProTox-II. Figure S1: molecule structure of plant-derived alkaloids. [file 7299778.f1.docx]

**Potential Therapeutic Applications of Plant-derived Alkaloids against Inflammatory and Neurodegenerative Diseases**

Babita Aryal^1,†^, Bimal Kumar Raut^1,†^, Salyan Bhattarai^2^, Sobika Bhandari^1^, Parbati Tandan^1^, Kabita Gyawali^1^, Kabita Sharma^1^, Deepa Ranabhat^1^, Ranjita Thapa^1^, Dipa Aryal^1^, Atul Ojha^3^, Hari Prasad Devkota^4^, and Niranjan Parajuli^1*^

*^1^Central Department of Chemistry, Tribhuvan University, Kirtipur, Kathmandu, Nepal*

*^2^Meakins-Christie Laboratories, Department of Medicine, McGill University, Montreal, Quebec, Canada*

*^3^Department of Chemistry and Chemical Biology, New Mexico University, Albuquerque, USA*

*^4^Graduate School of Pharmaceutical Sciences, Kumamoto University, 5-1 Oe-honmachi, Kumamoto 862-0973, Japan*

**Table S1** Plant-derived alkaloids with their biosynthetic precursors and current research

| **Class** | **Alkaloids** | **Current research** | **Precursors** | **Sources** |
| --- | --- | --- | --- | --- |
| Indole | Hunterines A-C | Cytotoxic activities were observed against HepG2, MCF-7, and A-549 human cancer cell lines using the MTT method |  Tuboxenine    Difforlemenine | <https://doi.org/10.1021/acs.joc.9b01835> |
|  | Erchinines A and B | Exhibited significant antimicrobial activity against *Trichophyton rubrum* and *Bacillus subtilis*, and compared with first-line antifungal drug griseofulvin and antibiotic cefotaxime. | Ibogaine | https://doi.org/10.1021/acs.orglett.8b01675 |
|  | Voacafricines A and B | Exhibited significant antimicrobial activities against *Staphylococcus aureus* and *Salmonella typhi,* and found their activities were superior to other known antibacterial drugs berberine and fibrauretine. |   19-epi-voacristine | <https://doi.org/10.1021/acs.orglett.8b00913> |
|  | Neocadambine A-D | It possesses anti-inflammatory and analgesic activities towards carrageenan-induced paw edema and acetic acid-induced animal models. |   Nauclechine | <https://doi.org/10.1016/j.jep.2020.113103> |
|  | Rauvomines A and B, Peraksine Alstoyunine | Rauvomines B exhibit remarkable inhibition of 264.7 macrophages with an IC_50_ value of 39.6 mM showing the anti-inflammatory activity and alstoyunine showed weak cytotoxicities toward HT- 29 and SWH80. |   Tryptophan    Secologanin | <https://doi.org/doi:10.1021/acs.orglett.7b01723>  <https://doi.org/10.1021/acs.jnatprod.5b00051> |
| Isoquinoline and its derivatives | Baicalensines A and B | Baicalensines A showed moderate cytotoxicity against the Caco-2 and HL-60 cell lines. | L-tyrosine    3-hydroxyphenyl acetaldehyde | <https://doi.org/10.1080/14786419.2020.1739040> |
|  | Hendersine A and B | Hendersine A and B showed a remarkable protective effect against H9C2 myocyte injuries induced by LPS-stimulation | L-tyrosine | <https://doi.org/10.1016/j.tetlet.2016.09.064> |
|  | Colchicine A | For [post-MI PET scan imaging of inflammation](https://clinicaltrials.gov/ct2/show/NCT02281305?term=Colchicine&cond=inflammation&draw=2&rank=8), it is in phase 4 and for inflammation, in acute coronary syndrome and vascular inflammation, it is in phase 3. Likewise, [colchicine for ALS](https://clinicaltrials.gov/ct2/show/NCT03693781?term=Colchicine&cond=Neurodegenerative+Diseases&draw=2&rank=1) is in phase 2. In 1682 patient months (mean follow-up, 20 months), treatment with colchicine significantly decreased the recurrence rate of Pericarditis. |  L-tyrosine   L-phenylalanine    Autumnaline | <https://doi.org/10.1007/978-3-642-22144-6_16>  <https://doi.org/10.1007/978-3-642-45410-3_24>  <https://clinicaltrials.gov/> |
| Indolidizine | Flueggeacosines A-C | Flueggeacoisine B exhibited potent activity in enhancing the neuronal differentiation of Neuro-2a cells. |   Securinine | <https://doi.org/10.1021/acs.orglett.8b03432> |
|  | Fluvirosaones A and B | Fluvirosaones A and B exhibit a weak effect in inhibiting triacylglycerides accumulation in 3T3-L1 cells. |   Virosecurinine |  |
|  | Norsecurinamines A and B |  |  N-methyltryptamine    Norsecurinine | <https://doi.org/10.1016/j.tetlet.2016.06.113> |
| Imidazole | Camellimidazole A−C, | Camellimidazole A and B exhibit significant protection against H_2_O_2_ -induced neuronal damage. |   Caffeine | <https://doi.org/10.1021/acs.orglett.8b00878> |
|  | Persicaline | With IC_50_ values of 0.1, 0.08, and 0.09 µM, persicaline has shown antioxidant activity. | Histidine betaine derivative | <https://doi.org/10.3390/molecules23020483> |
|  | Pilocarpine | (0.75% phentolamine ophthalmic Solution) Pilocarpine eye drops in subjects with presbyopia are in phase 2 clinical trials. |   L-histidine | [**https://doi.org/10.1007/978-3-642-22144-6_27**](https://doi.org/10.1007/978-3-642-22144-6_27)  [**https://clinicaltrials.gov/**](https://clinicaltrials.gov/) |
| Tropane | Cocaine  scopolamine/hyoscyamine  Calystegine | Scopolamine along with the combination of other drugs used in the treatment of Alzheimer's disease is in phase 1 clinical trials. |   L-ornithine   L-arginine | <https://doi.org/10.3390/molecules24040796> <https://clinicaltrials.gov/> |
| Pyridine | Nicotine/ anabasine | In the treatment of Parkinson's disease nicotine drug is the phase 1 and phase 2 clinical trial. |   Ornithine   Arginine | <https://doi.org/10.3390/molecules24040796>  <https://clinicaltrials.gov/> |
|  | Alangiumines A-C | Alangiumines A showed selective antitumor activity against glioma stem cells with IC_50_ values of 12.8 and 23.0 mM. |   Anabasine | [**https://doi.org/10.1016/j.tetlet.2019.151502**](https://doi.org/10.1016/j.tetlet.2019.151502) |
| Piperidine | Piperine | Administration of piperine in rats (20 mg/kg) increased the serum concentration whereas in humans 20 mg produced much higher concentrations from 0.25 to 1 h post-drug indicates piperine enhances the serum concentration, extent of absorption, and bioavailability in both rats and humans with no adverse effects. |  Phenylalanine    Lysine | <https://doi.org/10.3390/plants10010128>  [**https://doi.org/10/cjkvjn**](https://doi.org/10/cjkvjn) |
|  | PyracyclumineA-J | Pyracyclumine A-J possesses a weak inhibitory effect on menin - mixed lineage leukemia 1 (MLL1) protein - protein interaction. |  4,6,6-trimethyl-5,6-dihydro-2(1H)- pyridone,   3,5,5-trimethyl-1,5-dihydro-2H-pyrrol-2- one | [**https://doi.org/10.1021/acs.jnatprod.8b00239**](https://doi.org/10.1021/acs.jnatprod.8b00239) |
|  | Euphococcinine, cis-pinidine | Euphococcinine showed a weak activity against gram-negative bacteria. |   1-2 dehydropinidinone | [**https://doi.org/10/f5v5jt**](https://doi.org/10/f5v5jt) |
| pyrrolizidine | Penibruguieramine A |  | Proline-pentaketide-amide | [**https://doi.org/10.1021/ol5001523**](https://doi.org/10.1021/ol5001523) |
|  | Senecionine | Senecionine was metabolized faster in sheep than cattle. |  L-arginine,    L-isoleucine | <https://doi.org/10/c58hvb>  [**https://doi.org/10.3390/molecules24030498**](https://doi.org/10.3390/molecules24030498) |

**Table S2** Occurrence, pharmacology, and toxicity of alkaloids

| **Plant Source** | **Alkaloids** | **Reported pharmacological activities** | **Reported Toxicity** | **Sources** |
| --- | --- | --- | --- | --- |
| *Stephania rotunda*  *Corydalis* sp.  *Rauvolfia serpentina* | L-tetrahydropalmatine **(1)**  Fissisaine **(14)**  Isocorydine **(15)**  Salutaridine **(16)**  Rotundine **(26)** Cepharanthine **(27)**  Dehydroroemerine **(30)** | Antibacterial, antimalarial, antiviral, treatment of septic shock, anti-inflammatory, hypoglycemic activity, anti-osteoporosis, antiallergic, antioxidative, anticancer, neuroprotective, antipsychotic, and antimalarial |  | <https://doi.org/10.1002/ptr.4710> <https://doi.org/10.1016/j.jep.2007.02.005> <https://doi.org/10.1016/j.jep.2014.04.024> <https://doi.org/10/bkkx6w> <https://doi.org/10.1021/np50038a038> |
| *Berberis vulgaris, Berberis candidula*  *Coptis chinensis* | Berberine **(2)** | Anti-inflammatory, antidiabetics, antioxidant, and anti-Alzheimer | Diarrhea , constipation , flatulence and abdominal complaint | <https://doi.org/10.1021/np50038a038> <https://doi.org/10.1021/acs.jnatprod.8b00592>  <https://doi.org/10/gh5g55>  <https://doi.org/10.2174/1872213X12666180115153635> |
| *Stephania tetrandra* | Tetrandrine **(3)** | Cancer chemotherapy, anti-inflammatory, and antirheumatic treatment | Pulmonary toxicity | <https://doi.org/10/gkd2vj> |
| *Sophora alopecuroides* | Aloperine **(4)** | Anti-inflammatory, antitumor, and neuroprotective |  | <https://doi.org/10.3389/fphar.2020.538137> |
| *Sinomenium acutum* | Sinomenine **(5)** | Anti-inflammatory, and anti-endotoxin |  | <https://doi.org/10.1111/jvp.12807> |
| *Sophora alopecuroides* | Oxymatrine **(6)** | Anticancer, cancer suppression, antibacterial, anti-inflammatory, pain killing, asthma cough, and antitumor | Dizziness, headache, nausea, vomiting, palpitations, irritability, pallor | <https://doi.org/10/gkd2wz> <https://doi.org/10.1177%2F1721727X16642779> |
| *Peganum harmala* | Harmine **(7)** | Parkinson’s disease, nervosity, analgesic, antiproliferative, antineoplastic, insecticidal, antidiabetic, and antiviral | Headache, dizziness, nausea | <https://doi.org/10.1016/S0254-6272(15)30016-9>  <https://doi.org/10.1021/acs.orglett.0c02650> |
| *Crinum jagus* | Galantamine **(8)** | Antiacetylcholinesterase activity, and cytotoxicity |  | <https://doi.org/10.1016/j.phytochem.2020.112390> |
| *Nandina domestica* | Didehydroglaucine **(9)** | Treatment of human ailments such as asthma, whooping cough, pharyngeal tumors, and uterine bleeding | Pulmonary edema, congestion and hemorrhage in birds | <https://doi.org/10.1080/14786419.2014.921166>  <https://doi.org/10.4061/2010/818159> |
| *Meconopsis simplicifolia* | Simplicifolianine **(10)** | Antimalarial and antiplasmodial |  | <https://doi.org/10.1016/j.jep.2013.09.052> |
| *Alstonia macrophylla* | 10-DemethoxyvincorineN(4)-oxide **(11)** | Antiplasmodial activity |  | <https://doi.org/10.1016/j.phytochem.2013.11.014> <https://doi.org/10.1016/S0031-9422(00)84812-8> |
| *Atropa belladonna* | Atropine **(12)** | Anticholinergic and treatment for vomiting, nausea |  | <https://doi.org/10.1021/acschemneuro.8b00615> |
| *Sophora tonkinensis* | Sophtonseedline B **(13)**  Alopecurin A **(20)**  9α-hydroxysophocarpine **(21)** | Antiviral, insecticidal |  | <https://doi.org/10.1021/acs.jafc.0c06032> |
| *Actinodaphne macrophylla* | Reticuline **(17)** | Antiplasmodial activity antioxidant and anti-inflammatory |  | <https://doi.org/10.1177%2F1934578X1501000913> |
| *Actinodaphne macrophylla* | Bicuculine **(18)** | Antiplasmodial activity antioxidant and anti-inflammatory |  | <https://doi.org/10.1177%2F1934578X1501000913> |
| *Flueggea virosa* | Bubbialidine **(19)** | Anti-HIV, anti-inflammatory, and antimalarial |  | <https://doi.org/10/gkd2wd> |
| *Protulaca oleracea* | Oleracone **(22)** | Anti-inflammatory, anticholinesterase, and antioxidant |  | <https://doi.org/10.1021/acs.jafc.6b02673> <https://doi.org/10.1080/14786419.2020.1739040> <https://doi.org/10.1016/j.fitote.2019.05.005> |
| *Annona cherimola* | Pronuciferine **(23)** | Antioxidant, antimicrobial activities,  and anti-cancer | Blindness, gastrointestinal disturbances such as nausea, vomiting, flatulence, and atropine-like effects, including photophobia and dryness of the mouth | <https://doi.org/10.1016/S0031-9422(00)00486-6> <https://doi.org/10.3390/toxins11090506> <https://doi.org/10.1016/j.bjp.2015.07.006> |
| *Trigonella foenum-graecum* | Trigonelline **(24)** | Hypoglycemic, hypolipidemic, neuroprotective, antimigraine, sedative, memory-improving, antibacterial, antiviral, and anti-tumor activities | Teratogenic effects, deleterious toxic effects on reproductive performance | <https://doi.org/10.2174/092986712801323171> <https://doi.org/10.1016/j.jep.2010.06.033> |
| *Lindera aggregata* | Pallidine **(25)** | Treatment of hyperlipidemia and anti-microbial activity |  | <https://doi.org/10.1177%2F1934578X0900400111> <https://doi.org/10.1016/j.tetlet.2013.10.126> |
| *Hippeastrum vittatum* | Ismine **(28)**  Montanine **(29)** | Analgesis, diuretic, anti tumor , antiasthma and anti-inflammatory antiviral, anxiolytic, antidepressant and anticonvulsant, antitumoral | palpitation, vomiting, nausea , arrhythmaia shock, dizziness, hypertension and comma violent body tremors and clonic convulsions | <https://doi.org/10.1016/j.fitote.2010.11.001> <https://doi.org/10.1016/j.pbb.2006.07.027> <https://doi.org/10.1021/np0005816> |
| *Papavier somniferum* | Morphine **(31)** Codeine **(32)** Thebaine **(33)** Papaverine **(34)** Narcotine **(35)** | Analgesic, anticarcinogenic, antimicrobial, antimutagenic, and antiparasitic | Coronary artery disease | <https://doi.org/10.1016/B978-0-08-100085-4.00006-2> <https://doi.org/10/c6dnp9> <https://doi.org/10.1007/s11101-018-9563-3> <https://doi.org/10.1016/j.indcrop.2012.04.018> |
| *Lycoris radiata* | Lycorine **(36)**  Lycoranine A **(37)** Lycoranine B **(38)** (+)-5,6-dehydrolycorine **(39)**  2α-methoxy-6-O-ethyloduline **(40)** O-demethyllycoramine N-oxide **(41)**  (+)-8,9-methylenedioxyl-homolycorine-N-oxide **(42)**  N-chloromethyl ungiminorine **(43)**  (+)-homolycorine- N-oxide **(44)** | Anti-inflammatory, antiviral, antibacterial, antitumor, antimalarial, cytotoxic |  | <https://doi.org/10/f5cpfv> <https://doi.org/10.3390/molecules18032458> <https://doi.org/10.1016/j.fitote.2013.05.006> <https://doi.org/10.1248/cpb.57.610> |
| *Peganum harmala* | Harmaline **(45)**  Pegaharine A **(46)** Pegaharine B **(47)** Pegaharine C **(48)**  Pegaharines D **(49)** Pegaharine E **(50)** Pegaharine F **(51)** | Parkinson’s disease, nervosity, analgesic, antiproliferative, antineoplastic, insecticidal, antidiabetic, and antiviral | Headache, dizziness, nausea | <https://dx.doi.org/10.4103%2F0973-7847.120524> <https://doi.org/10.1021/acs.orglett.0c02650> |
| *Catharanthus roseus* | Vindoline **(52)**  Vindolidine **(53)**  Vindolicine **(54)**  Vindolinine **(55)**  Cathachunine **(56)**  Vinblastine **(57)** Vincristine **(58)** | Antidiabetic, antioxidant  antitumour |  | <https://doi.org/10.3389/fpls.2019.00931> <https://doi.org/10.3390/molecules18089770> |
| *Psychotria colorata* | Hodgkinsine **(59)** Psychotridine **(60)** | Analgesic |  | <https://doi.org/10/dr3nc7> <https://doi.org/10.1016/0378-8741(95)01287-N> |
| *Camptotheca acuminata* | Camptothecin **(61)** | Anticancer and antiviral |  | <https://doi.org/10/fkrxmg> |
| *Crinum latifolium* | 6-methoxyundulatine **(62)** 6-methoxycrinamidine **(63)**  Undulatine N-oxide **(64)**  Augustmanine **(65)**  Perlolyrine **(66)** | Antipyretics, diuretics, antiasthmatics, antimalarial, antiaging, antitumor |  | <https://doi.org/10.1016/j.phytol.2018.01.004> |
| *Sida acuta* | Quinodolinone **(67)** Cryptolepine **(68)** Cryptolepinone **(69)**  11-methoxyquindoline **(70)**  Vasicinone **(71)**  Vasicine **(72)** | Antimalarial  Cytotoxic/anticancer  Antimicrobial |  | <https://doi.org/10.3390/medicines4040075><https://doi.org/10.3390/molecules18032769><https://doi.org/10/b9b4fb> |
| *Berberis vulgaris , Berberis candidula* | Bersavine **(73)** Berbidine **(74)**  Berbamine  **(75)** Obamegine (**76)** Muraricine **(77)** Aromoline **(78)** Berbostrejdine **(79)** | Anti-inflammatory, antidiabetics, and antioxidant  Anti-Alzheimer | diarrhea , constipation , flatulence and abdominal complaint | <https://doi.org/10.3390/ijms14022928> <https://doi.org/10.1021/acs.jnatprod.8b00592> <https://doi.org/10/gh5g55> <https://doi.org/10.2174/1872213X12666180115153635> |
| *Menispermum dauricum* | Dauricine **(80)** | Anti-inflammatory and anticancer | pulmonary injury | <https://doi.org/10.1016/j.abb.2019.03.018> |
| *Pseuduvaria setosa* | N-methylouregidione **(81)** Ouregidione **(82)** Liriodenine **(83)** Oxostephanine **(84)** | Antituberculosis, antimalarial, anticancer, and immunomodulatory |  | <https://doi.org/10.1080/13880200600714111> |
| *Annona cherimola* | Anonaine **(85)** Asimilobine **(86)** Lanuginosine **(87)**  Cherianoine **(88)**  Lysicamine **(89)** Stepharine **(90)** | Antioxidant, antimicrobial activities, anticancer | blindness, gastrointestinal disturbances such as nausea, vomiting, flatulence, and atropine-like effects, including photophobia and dryness of the mouth | <https://doi.org/10.1016/S0031-9422(00)00486-6><https://doi.org/10.3390/toxins11090506><https://doi.org/10.1016/j.bjp.2015.07.006> |
| *Mahonia aquifolium* | Jatrorrhizine **(91)** | Antimicrobial, antitumoral, and Immunomodulatory |  | <https://doi.org/10.1155/2019/6439021> <https://doi.org/10.1002/ptr.1517> |
| *Fritillaria ussuriensis* | Verticinone **(92)**  Verticine **(93)**  Peimisine **(94)** | Anti-Inflammatory, antitussive, antiasthmatic and antihypertensive |  | <https://doi.org/10/b7gnch> |
| *Lindera aggregata* | (+)-norboldine acetate **(95)**  (+)-norboldine **(96)**  (+)-boldine **(97)**  (+)-laurotetanine **(98)**  (+)-N-methyl laurotetanine) **(99)** Linderaggrine A **(100)** | Treatment of hyperlipidemia  antimicrobial activity |  | <https://doi.org/10.1177%2F1934578X0900400111> <https://doi.org/10.1016/j.tetlet.2013.10.126> |
| *Sophora alopecuroides* | Matrine **(101)** Sophoridine **(102)** | Anticancer suppression, antibacterial, anti-inflammatory, pain killing, asthma cough, and antitumor | Dizziness, headache, nausea, vomiting, palpitations, irritability, and pallor | <https://doi.org/10/gkd2wz> <https://doi.org/10.1177%2F1721727X16642779> |
| *Hippeastrum vittatum* | Aconitine **(103)** Vittacarboline **(104)**  O-methylismine **(105)** | Analgesis, diuretic, antitumor , antiasthma and anti-inflammatory Antiviral, anxiolytic, antidepressant, anticonvulsant, and antitumoral | Palpitation, vomiting, nausea, arrhythmia shock, dizziness, hypertension and comma violent body tremors, and clonic convulsions | <https://doi.org/10.1016/j.fitote.2010.11.001><https://doi.org/10.1016/j.pbb.2006.07.027><https://doi.org/10.1021/np0005816> |
| *Sceletium species* | Δ^7^mesembrenone **(106)** Mesembranol **(107)** Mesembrenone **(108)** Mesembrine **(109)** Epimesembranol **(110)** | Antipsychotic, antianxiety, anti-depressant |  | <https://doi.org/10.1016/j.jep.2011.07.035><https://doi.org/10.18433/J3DK5F> |
| *Stephania rotunda* | Pseudopalmatine **(111)** Oxoxylopine **(112)** Stepharine **(113)** Tuduranine **(114)** Thalifoline **(115)** Cepharanoline **(116)** Coclaurine **(117)** Dehassiline **(118)** Fangchinoline **(119)** Polycarpine **(120)** Palmatrubine **(121)** Jatorrhizine **(122)** Columbamine **(123)** Dehydrocorydalmine **(124)**  Stepharotudine **(125)** Palmatine **(126)**  (-)-Thaicanine N-oxide (4- hydroxycorynoxidine **(127)**  Corynoxidine **(128)** Xylopinine **(129)** Roemerine **(130)** Liriodernine **(131)** Stephanine **(132)** Vireakine **(133)** Lanuginosine **(134)** | Antibacterial, antimalarial, antiviral, treatment of septic shock, anti-inflammatory, hypoglycemic activity, antiosteoporosis, antiallergic, antioxidative, anticancer, neuroprotective, antipsychotic, and antimalarial |  | <https://doi.org/10.1002/ptr.4710> <https://doi.org/10.1016/j.jep.2007.02.005> <https://doi.org/10.1016/j.jep.2014.04.024> <https://doi.org/10/bkkx6w><https://doi.org/10.1021/np50038a038> |
| *Ficus septica* | Dehydrotylophorine **(135)** Dehydroantine **(136)** Tylophoridicine D **(137)** | Antiinflammatory antitumor, antimalarial antifungal, and antibacterial activities |  | <https://doi.org/10.1248/cpb.c16-00181> |
| *Selaginella repanda* | 8-hydroxyquinoline **(138)** Norharman **(139)** Hordenine **(140)** | Antimalarial, antioxidant, antibacterial, antiviral, anticancer and anti-inflammatory. |  | <https://doi.org/10.3390/plants6040042> |
| *Actinodaphne macrophylla* | Cycleanine **(141)**  10-demethylxylopinine **(142)**  Laurotetanine **(143)**  α-hydrastine **(144)** Parfumine **(145)**  Anolobine **(146)** | Antiplasmodial activity antioxidant and anti-inflammatory |  | <https://doi.org/10.1177%2F1934578X1501000913> |
| *Flueggea virosa* | Flueggenine E **(147)** Flueggenine A **(148)** Flueggenine B **(149)** Norsecurinic acid **(150)** 15β-Butoxy-14,15-dihydronorsecurinine **(151)**  15α-Butoxy-14,15-dihydronorsecurinine **(152)**  (−)-norsecurinine **(153)** Niruroidine **(154)** Flueggenine C **(155)** FlueggeninnG **(156)** Flueggeninn H **(157)** Fluevirosine F **(158)** Fluevirosine G **(159)** Fluevirosine E **(160)** Bergenin **(161)**  Flueggether A **(162)** Virosinine A **(163)** | Anti-HIV, anti-inflammatory, and antimalarial |  | <https://doi.org/10/gkd2wd><https://doi.org/10.1177%2F1934578X0600101002> <https://doi.org/10.1021/acs.jnatprod.9b00142> <https://doi.org/10.1016/j.biopha.2017.02.056> <https://doi.org/10.1021/ol303146a> |
| *Securinega suffruticosa* | 7a-(pyridin-2-yl)-7,153.7adihydrobenzofuran-2(6H)-one **(164)** Securinine **(165)** Securitinine **(166)**  4-epiphyllanthine **(167)** Menisdaurilide **(168)** | Cytotoxic, anti-inflammatory, neuroprotective |  | <https://doi.org/10.1021/acs.jnatprod.9b00142> |
| *Stephania epigea* | Epigasine A **(169)** Epigasine B **(170)** Pronuciferine **(171)** Dehydrodicentrine **(172)** Romeline **(173)**  Romerine **(174)**  Dicentrine **(175)**  N-methylcalycinine **(176)** N-methyllaurotetanine **(177)**  Phanostenine **(178)**  3′-nor-4′-oxo-cepharanthie **(179)** | Anti-Alzheimer and  cytotoxicity |  | <https://doi.org/10.1016/j.fitote.2015.05.019><https://doi.org/10.1021/np400084t> |
| *Protulaca oleracea* | Oleracimine **(180)** Oleracimine A **(181)** Oleraciamid G **(182)** Oleraindole D **(183)** Oleraindole B **(184)** Olerindole A **(185)** Neochinulin D **(186)** Neoechinulin A **(187)** Echinulin **(188)** | Anti-inflammatory, anticholinesterase and  antioxidant |  | <https://doi.org/10.1021/acs.jafc.6b02673><https://doi.org/10.1080/14786419.2020.1739040> <https://doi.org/10.1016/j.fitote.2019.05.005> |
| *Flindersia pimenteliana* | Pimentelamines A **(189)**  Pimentelamin B **(190)** Pimentelamin C **(191)** | Antiplasmodial |  | <https://doi.org/10.1021/acs.jnatprod.7b00587> |
| *Crinum delagoense* | 6-Hydroxycrinamine **(192)**  Hamayne **(193)** | Anticancer |  | <https://doi.org/10.1002/cmdc.201100608> <https://doi.org/10.1016/S0031-9422(98)00245-3> |
| *Rauwolfia serpentina* | Reserpine **(194)** | Hypertension, tachycardia, and thyrotoxicosis, | Lethargy, sedation, psychiatric depression, hypotension, nausea, vomiting, abdominal cramping, gastric ulceration, nightmares, skin rash, and itching, galactorrhea, breast enlargement, sexual dysfunction, and withdrawal psychosis in 1 case. | <https://www.ncbi.nlm.nih.gov/pmc/articles/PMC4566472/> |
| *Tabernaemontana catharinensis* | Voacangine **(195)** Heyneanine **(196)** Coronaridine **(197)** | Antioxidant activity, anticholinesterase activity, antitumor activity |  | <https://doi.org/10.1155/2013/519858> <https://doi.org/10.1590/S1415-47572013005000010> |
| *Aconitum laciniatum* | Pseudaconitine **(198)** Neoline **(199)**  Senbusine A **(200)** | Anti-inflammatory, and antibacterial, |  | <https://doi.org/10/f7mnc6> |
| *Stephania cepharantha* | (6S,7S,9R,13S)N-formylsinococuline **(201)** (6S,7S,9R,13S)-6,7-di-O-acetyl-N-formylsinococuline **(202)** (6S,7S,9R,13S)-6-O-acetyl-N-formylsinococuline **(203)**  (6S,7S,9R,13S)-7-O-acetyl-N-formylsinococuline **(204)** (1R,1′S)-N-formylcepharanthine **(205)** | Anti-neuroinflammatory |  | <https://doi.org/10.1021/acs.jnatprod.9b00483> |
| *Areca catechu* | Guvacine **(206)**  Arecoline **(207)**  Choline **(208)**  Arcaine **(209)** | Antiulcerogenic, antioxidant, anticonvulsant, central nervous system stimulant activity, antifertility, oxytocic activity, antiviral activity, and anthelmintic | Pre-cancerous oral lesions as well as oral and esophageal cancers | <https://doi.org/10.1590/S1415-47572013005000010> <https://doi.org/10.1021/acs.jafc.6b05140> |
| *Physostigma venenosum* | Physostigmine **(210)** | Treatment of AD | Muscarinic receptor stimulation includes nausea, vomiting, diarrhea, abdominal pain, respiratory tract secretions, hypersalivation, seizures, Diaphoresis, Tachycardia, blurred vision, bradycardia, and atrial fibrillation | <https://doi.org/10.22270/jddt.v10i1-s.3866> |
| *Fruit of Nandina domestica Thunb* | Palmatine **(211)**O-Methylbulbocapnine **(212)** Dehydronantenine **(213)** Nantenine **(214)**  Glaucine **(215)** Didehydroglaucine **(216)** Dehydrocorydaline **(217)** Magnoflorine **(218)** | Treatment of human ailments such as asthma, whooping cough, pharyngeal tumours, and uterine bleeding | Pulmonary edema, congestion, and hemorrhage in birds | <https://doi.org/10.1080/14786419.2014.921166> <https://doi.org/10.4061/2010/818159> |
| *Sophora alopecuroides* | Sophocarpine **(219)** Sophoridine **(220)** | Antiviral against hepatitis B |  | <https://doi.org/10.1021/acs.orglett.8b02637> |
| *Stephania venosa* | Dehydrocrebanie **(221)** Stephanine **(222)** Crebanine **(223)**  O-methybulbocapine **(224)** | Antiplasmodial, and anticancer |  | <https://doi.org/10.1002/ptr.5861> |
| *Meconopsis simplicifolia* | Protopine **(225)**  Norsanguinarine **(226)**  Dihydrosanguinarine **(227)**  6-methoxydihydrosanguinarine **(228)**  Oxysanguinarine **(229)** | Antimalarial and antiplasmodial |  | <https://doi.org/10.1016/j.jep.2013.09.052> |
| *Alstonia macrophylla* | Alstofolinine A **(230)**  20,21-Dihydroalstonerine **(231)**  Macrocarpine D **(232)**  Macrodasine H **(233)**  Alstonoxine C **(234)**  Alstonoxine D **(235)**  19,20-Z-Affinisine **(236)**  10-Demethoxyvincorine **(237)**  11-Methoxyvincorine **(238)**  11-Demethoxyquaternine **(239)**  Talcarpine **(240)**  Vincamajine **(241)**  Vincorine **(242)**  Cabucraline **(243)** | Antiplasmodial activity |  | <https://doi.org/10.1016/j.phytochem.2013.11.014><https://doi.org/10.1016/S0031-9422(00)84812-8> |
| *Atropa belladonna* | Scopolamine **(244)** | Anticholinergic  And treatment for vomiting, nausea |  | <https://doi.org/10.1021/acschemneuro.8b00615> |
| *Strychnos icaja* | Strychnogucine B **(245)** | Antimalarial, and antitrypanosomal activity |  | <https://doi.org/10/gdrrjm> |
| *Strychnos nigritana* | Nigritanine **(246)** | Antimicrobial, and cytotoxic activity |  | <https://doi.org/10.3390/toxins11090511> |
| *Sophora tonkinensis* | Sophtonseedline A **(247)** Sophtonseedline C **(248)** Sophtonseedline D **(249)** Sophtonseedline E **(250)** Sophtonseedline F **(251)** Sophtonseedline G **(252)** Sophtonseedline H **(253)** Sophtonseedline I **(254)** Sophtonseedline J **(255)** Sophtonseedline K **(256)** 5α-hydroxysophocarpine **(257)**  Sophoranol **(258)**  9α-hydroxymatrine **(259)**  5α-9α-dihydroxymatrine **(260)**  6,7- dehydro-matrine **(261)**  5-hydroxy-6.7-dehydro-matrine **(262)**  5.6-dehydro-matrine **(263)**  Cytisine **(264)**  N-methylcytisine **(265)** N-formylcytisine **(266)**  N-acylcytisine **(267)** | Antiviral, and insecticidal |  | <https://doi.org/10.1038/aps.2016.125> |
| *Crinum jagus* | Sanguinine **(268)**  Cherylline **(269)**  Crinine **(270)**  Hippadine **(271)** | Anti-acetylcholinesterase activity, and cytotoxicity |  | <https://doi.org/10.1016/j.phytochem.2020.112390> |
| *Chelidonium majus* | Chelidonine **(272)**  Chelerythrine **(273)**  Sanguinarine **(274)**  Coptisine **(275)**  Allocryptopine **(276)**  Protopine **(277)** | Antibacterial and antifungal |  | <https://doi.org/10.3390/toxins11070406> |
| *Coffea arabica* | Caffeine **(278)** | Anti-inflammatory, and anti-neurodegenerative |  | <https://doi.org/10/gg7zq2> <https://doi.org/10.1111/cns.12684> |
| *Salsola oppositofolia* | Salsoline **(279)** | Anti-alzheimer's |  | <https://doi.org/10.1111/cns.12684> |
| *Piper nigrum* | Piperine **(280)** | Anti-inflammatory, Antidepressant |  | <https://doi.org/10.1007/978-3-319-41334-1_8> |

**Table S3** ADMET properties of promising plant alkaloids by pkCSM server

| Alkaloids | Absorption | | | | Distribution | | | Metabolism (Inhibitor) | | | | | Excretion | | Toxicity | |
| --- | --- | --- | --- | --- | --- | --- | --- | --- | --- | --- | --- | --- | --- | --- | --- | --- |
|  | Water solubility (log mol/L) | Caco2 permeability (log Papp 10-6 cm/s) | Intestinal absorption (% absorbed) | Skin permeability (log Kp) | VDss (Human, log L/Kg) | BBB Permeability (logBB) | CNS Permeability (log PS) | CYP1A2 | CYP2C19 | CYP2C9 | CYP2D6 | CYP3A4 | Renal OCT substrate | Total clearance (log ml/min/kg) | AMES toxicity | Hepatotoxicity |
|  |  |  |  |  |  |  |  |  |  |  |  |  |  |  |  |  |
| Nicotine | -0.87s | 1.671 | 95.867 | -2.144 | 0.595 | 0.208 | -2.995 | YES | NO | NO | NO | NO | NO | 0.86 | NO | YES |
| 1 | -3.51 | 1.042 | 93.004 | -2.891 | 0.862 | 0.081 | -1.397 | NO | NO | NO | YES | NO | YES | 1.149 | NO | NO |
| 2 | -3.67 | 2.024 | 99.963 | -2.63 | 0.671 | 0. 533 | -1.573 | YES | NO | NO | YES | NO | NO | 1.276 | NO | NO |
| 3 | -3.93 | 1.13 | 96.63 | -2.73 | -0.85 | -1.05 | -2.47 | NO | NO | NO | NO | NO | YES | 0.685 | YES | NO |
| 4 | -2.39 | 1.35 | 94.744 | -2.528 | 1.317 | 0.909 | -2.936 | NO | NO | NO | NO | NO | NO | 0.769 | NO | NO |
| 5 | -3.30 | 1.245 | 95.116 | -3.827 | 0.769 | -0.682 | -2.355 | NO | NO | NO | NO | NO | YES | 0,958 | YES | YES |
| 6 | -3.29 | 1.171 | 95.111 | -2.736 | 1.293 | -0.699 | -2.018 | NO | YES | NO | NO | YES | NO | 0.849 | NO | NO |
| 7 | -3.37 | 1.79 | 93.67 | -2.64 | 0.18 | 0.36 | -1.62 | YES | NO | NO | NO | NO | NO | 0.6 | YES | NO |
| 8 | -2.27 | 1.75 | 93.571 | -3.032 | 0.891 | 0.498 | -2.957 | NO | NO | NO | NO | NO | NO | 0.951 | NO | YES |
| 9 | -4.615 | 0.398 | 97.949 | -2.735 | -0.816 | -1.554 | -2.439 | NO | NO | NO | NO | NO | NO | 0.121 | NO | NO |
| 10 | -3.597 | 1.251 | 98.604 | -2.774 | 0.64 | -0.705 | -2.885 | YES | NO | NO | NO | NO | NO | 1.352 | NO | NO |
| 11 | -4.527 | 1.15 | 98.338 | -3.077 | 0.693 | 0.074 | -1.969 | NO | YES | NO | NO | NO | NO | 0.528 | NO | NO |
| 12 | -2.183 | 1.229 | 93.077 | -2.821 | 0.934 | 0.642 | -2.463 | NO | NO | NO | NO | NO | YES | 1.036 | NO | NO |
| 13 | -2.795 | 0.35 | 62.36 | -3.772 | 0.355 | -0.189 | -3.517 | NO | NO | NO | NO | NO | NO | 0.709 | NO | NO |
| 14 | -3.571 | 1.112 | 93.064 | -2.809 | 0.825 | -0.361 | -2.243 | YES | NO | NO | NO | NO | NO | 1.195 | NO | NO |
| 15 | -3.499 | 0.955 | 92.521 | -2.771 | 1.342 | 0.226 | -2.109 | YES | NO | NO | YES | NO | NO | 0.961 | NO | NO |
| 16 | -2.32 | 1.182 | 95.019 | -3.41 | 0.868 | -0.067 | -2.37 | NO | NO | NO | NO | NO | NO | 1.003 | NO | NO |
| 17 | -3.481 | 0.918 | 92.241 | -2.745 | 0.978 | -0.322 | -2.354 | YES | NO | NO | YES | NO | NO | 1.005 | NO | NO |
| 18 | -2.998 | 1.32 | 94.994 | -3.19 | 0.611 | -0.719 | -2.893 | YES | YES | NO | NO | YES | NO | 0.964 | NO | NO |
| 19 | -2.38 | 1.169 | 85.108 | -3.924 | 0.691 | -0.214 | -3.078 | NO | NO | NO | NO | NO | NO | 1.271 | NO | NO |
| 20 | -3.091 | 0.599 | 65.923 | -4.127 | -0.106 | -0.51 | -3.492 | NO | NO | NO | NO | NO | NO | 1.195 | NO | NO |
| 21 | -2.282 | 1.177 | 77.487 | -3.737 | 0.665 | -0.077 | -3.594 | NO | NO | NO | NO | NO | NO | 0.602 | NO | NO |
| 22 | -4.036 | 1.308 | 94.334 | -3.258 | 0.045 | -0.057 | -2.853 | NO | NO | NO | NO | NO | NO | 0.976 | NO | NO |
| 23 | -2.4 | 1.662 | 94.366 | -2.971 | 0.934 | 0.182 | -2.997 | NO | NO | NO | YES | NO | NO | 0.998 | NO | NO |
| 24 | -1.627 | 1.12 | 96.37 | -2.744 | -0.599 | -0.232 | -2.727 | NO | NO | NO | NO | NO | NO | 0.378 | NO | NO |
| 25 | -3.019 | 0.669 | 91.717 | -2.735 | 1.495 | -0.94 | -2.939 | NO | YES | NO | NO | YES | NO | 1.2 | NO | NO |
| 26 | -3.512 | 1.042 | 93.004 | -2.891 | 0.862 | 0.081 | -1.397 | NO | NO | NO | YES | NO | YES | 1.149 | NO | NO |
| 27 | -3.779 | 0.942 | 95.709 | -2.735 | -0.739 | -0.786 | -2.435 | NO | NO | NO | NO | NO | NO | 0.691 | YES | NO |
| 28 | -3.482 | 1.176 | 93.664 | -2.825 | 0.212 | -0.028 | -2.198 | YES | YES | NO | NO | NO | NO | 0.223 | NO | NO |
| 29 | -2.031 | 1.587 | 96.581 | -2.315 | -0.14 | 0..032 | -2.29 | YES | NO | NO | NO | NO | NO | 0.773 | NO | NO |
| 30 | -5.553 | 1.59 | 98.77 | -2.543 | 0.661 | 0.339 | -1.452 | YES | YES | YES | NO | NO | NO | 0.252 | NO | NO |
| 31 | -2.16 | 1.15 | 81.41 | -2.85 | 0.89 | -0.15 | -2.42 | NO | NO | NO | YES | NO | NO | 0.81 | NO | YES |
| 32 | -2.09 | 1.84 | 94.26 | -2.92 | 1.11 | -0.01 | -2.86 | NO | NO | NO | YES | NO | NO | 0.83 | NO | YES |
| 33 | -2.29 | 1.87 | 94.21 | -2.96 | 1.04 | 0.37 | -2.91 | NO | NO | NO | YES | NO | YES | 1.12 | NO | NO |
| 34 | -4.63 | 1.41 | 97.56 | -2.53 | -0.07 | -0.47 | -2.18 | YES | YES | YES | NO | YES | YES | 0.29 | NO | YES |
| 35 | -3.59 | 1.39 | 93.57 | -2.78 | 0.23 | -0.67 | -3.14 | NO | YES | NO | NO | YES | YES | 0.91 | NO | NO |
| 36 | -2.19 | 0.59 | 88.88 | -2.98 | 0.46 | -0.23 | -2.65 | NO | NO | NO | NO | NO | NO | 0.99 | NO | YES |
| 37 | -4.09 | 1.33 | 98.89 | -2.52 | 0.04 | -0.07 | -2 | YES | YES | NO | NO | NO | YES | 0.41 | YES | NO |
| 38 | -4.29 | 1.36 | 98.8 | -2.53 | 0.09 | -0.06 | -1.93 | YES | YES | NO | NO | NO | YES | 0.4 | YES | YES |
| 39 | -1.89 | 0.65 | 88.04 | -3.22 | 0.51 | -0.19 | -2.53 | NO | NO | NO | NO | NO | NO | 1.17 | NO | NO |
| 40 | -2.951 | 1.134 | 95.431 | -3.441 | 0.739 | 0.05 | -2.41 | NO | NO | NO | NO | NO | YES | 0.904 | NO | YES |
| 41 | -3.796 | 1.002 | 94.691 | -3.866 | 0.059 | -0.025 | -2.217 | NO | NO | NO | NO | NO | NO | 0.705 | NO | NO |
| 42 | -4.08 | 1.61 | 97.72 | -3.38 | 0.18 | -0.39 | -2.81 | NO | NO | NO | NO | NO | NO | 0.45 | YES | NO |
| 43 | -2.44 | 0.726 | 88.124 | -2.94 | 0.436 | -0.534 | -2.684 | NO | NO | NO | NO | NO | NO | 0.949 | NO | YES |
| 44 | -4.14 | 1.18 | 97.32 | -3.34 | 0.13 | -0.45 | -2.79 | NO | NO | NO | NO | NO | NO | 0.6 | NO | NO |
| 45 | -3.18 | 1.57 | 93.47 | -2.44 | 0.29 | 0.34 | -2.12 | YES | NO | NO | NO | NO | NO | 0.57 | YES | NO |
| 46 | -2.39 | 0.58 | 86.83 | -2.9 | 0.21 | -0.32 | -2.27 | YES | NO | NO | NO | NO | NO | 0.32 | YES | NO |
| 47 | -3.95 | 1.28 | 95.23 | -2.92 | 0.25 | 0.07 | -2.79 | YES | YES | NO | NO | NO | NO | 0.49 | NO | YES |
| 48 | -4.27 | 0.85 | 94.63 | -2.75 | 0.33 | -0.47 | -1.73 | YES | YES | YES | NO | YES | YES | 0.4 | NO | YES |
| 49 | -3.85 | 0.79 | 96.05 | -2.74 | 0.34 | -0.37 | -1.57 | YES | YES | YES | NO | YES | NO | 0.1 | YES | YES |
| 50 | -4.43 | 0.74 | 93.78 | -2.74 | 0.34 | 0.45 | -1.41 | YES | YES | YES | NO | YES | NO | 0.54 | YES | NO |
| 51 | -4.29 | 0.74 | 94.25 | -2.74 | 0.36 | 0.39 | -1.24 | YES | YES | YES | NO | YES | NO | 0.62 | NO | NO |
| 52 | -3.48 | 0.32 | 99.48 | -3.28 | 0.6 | -0.24 | -3.39 | NO | NO | NO | NO | NO | NO | 0.36 | NO | NO |
| 53 | -3.22 | 0.23 | 99.15 | -3.42 | 0.77 | -0.05 | -2.69 | NO | NO | NO | NO | NO | NO | 0.36 | NO | NO |
| 54 | -3.45 | 1.19 | 97.84 | -2.73 | -0.47 | -1.57 | -3.28 | NO | NO | NO | NO | NO | NO | 0.27 | NO | YES |
| 55 | -3.69 | 1.16 | 98.23 | -3.16 | 1.27 | 0.36 | -2.84 | NO | NO | NO | NO | NO | NO | 0.44 | NO | YES |
| 56 | -3.24 | 1.25 | 97.62 | -2.74 | 0.95 | -1.06 | -3.08 | NO | NO | NO | NO | NO | NO | 0.55 | NO | NO |
| 57 | -3.06 | 0.23 | 100 | -2.74 | 1.32 | -1.41 | -3.73 | NO | NO | NO | NO | YES | NO | 0.29 | NO | YES |
| 58 | -3.03 | 0.18 | 100 | -2.74 | 1.34 | -1.59 | -3.92 | NO | NO | NO | NO | YES | NO | 0.41 | NO | YES |
| 59 | -2.89 | -0.38 | 85.09 | -2.74 | 0.79 | 0.24 | -0.97 | YES | NO | YES | YES | NO | YES | 0.92 | NO | NO |
| 60 | -2.89 | 3.01 | 86.7 | -2.74 | 0.01 | -0.47 | -1.67 | YES | NO | NO | NO | NO | YES | 0.54 | NO | NO |
| 61 | -3.22 | 1.24 | 99.51 | -2.85 | 0.19 | -0.17 | -2.18 | YES | NO | NO | NO | NO | YES | 0.62 | NO | YES |
| 62 | -2.38 | 1.21 | 93.25 | -3.09 | 0.54 | -0.52 | -2.47 | NO | NO | NO | NO | NO | NO | 1.25 | NO | NO |
| 63 | -2.32 | 0.7 | 93.18 | -2.95 | 0.57 | -0.25 | -2.57 | NO | NO | NO | NO | NO | NO | 1.22 | NO | NO |
| 64 | -3.76 | 0.99 | 97.5 | -3.17 | 0.03 | -0.61 | -2.47 | NO | NO | NO | NO | NO | NO | 0.69 | YES | NO |
| 65 | -1.91 | 1.74 | 94.87 | -2.91 | 0.87 | 0.42 | -3.05 | NO | NO | NO | YES | NO | YES | 1.16 | NO | YES |
| 66 | -3.92 | 0.72 | 95.59 | -2.74 | 0.81 | 0.43 | -1.91 | YES | YES | NO | NO | YES | NO | 0.46 | YES | NO |
| 67 | -4.25 | 1.24 | 94.2 | -2.77 | -0.02 | 0.46 | -1.44 | YES | YES | YES | NO | YES | NO | 0.46 | YES | NO |
| 68 | -4.53 | 1.52 | 97.48 | -2.09 | 0.62 | 0.28 | -1.47 | YES | YES | YES | NO | NO | NO | 0.92 | YES | NO |
| 69 | -4.41 | 1.37 | 96.23 | -2.72 | 0.02 | 0.54 | -1.33 | YES | YES | YES | NO | YES | NO | 0.49 | YES | NO |
| 70 | -3.95 | 1.846 | 92.112 | -2.808 | 0.326 | 0.464 | -1.432 | YES | YES | YES | NO | NO | NO | 0.767 | YES | NO |
| 71 | -2.47 | 1.189 | 90.602 | -3.034 | 0.059 | -0.205 | -2.81 | NO | NO | NO | NO | NO | NO | 0.566 | NO | NO |
| 72 | -2.22 | 1.576 | 93.261 | -2.708 | 0.183 | -0.192 | -2.568 | NO | NO | NO | NO | NO | NO | 0.572 | NO | NO |
| 73 | -2.97 | 0.658 | 87.718 | -2.735 | -0.32 | -1.223 | -2.485 | YES | NO | NO | NO | YES | NO | 0.83 | YES | NO |
| 74 | -3.95 | 1.173 | 94.506 | -2.839 | 0.985 | 0.015 | -2.5 | NO | NO | NO | NO | YES | NO | 0.93 | NO | NO |
| 75 | -3.23 | 0.956 | 93.59 | -2.735 | -1.123 | -1.121 | -2.409 | NO | NO | NO | NO | NO | NO | 0.74 | YES | NO |
| 76 | -3.13 | 0.822 | 92.224 | -2.735 | -1.221 | -1.103 | -2.45 | NO | NO | NO | NO | NO | NO | 0.735 | NO | NO |
| 77 | -3.96 | 0.848 | 91.265 | -2.738 | 1.075 | -0.076 | -2.202 | NO | NO | NO | YES | YES | NO | 1.014 | NO | NO |
| 78 | -3.09 | 0.707 | 90.888 | -2.735 | -0.951 | -1.063 | -2.44 | NO | YES | NO | NO | NO | NO | 0.706 | YES | NO |
| 79 | -3.15 | -0.029 | 93.78 | -2.735 | -1.082 | -1.259 | -2.841 | NO | YES | NO | NO | YES | NO | 0.912 | YES | NO |
| 80 | -3.55 | 0.485 | 92.345 | -2.735 | -0.337 | -1.09 | -2.574 | NO | YES | NO | YES | YES | NO | 1.021 | YES | NO |
| 81 | -4.96 | 1.252 | 98.718 | -2.653 | -0.022 | -0.492 | -2.147 | YES | YES | YES | NO | NO | NO | 0.172 | YES | YES |
| 82 | -4.03 | 1.302 | 95.338 | -2.814 | 0.003 | -0.467 | -2.195 | YES | YES | YES | NO | YES | NO | 0.065 | YES | YES |
| 83 | -3.88 | 1.316 | 100 | -2.652 | 0.033 | 0.031 | -1.641 | YES | YES | YES | NO | YES | NO | 0.107 | YES | YES |
| 84 | -4.02 | 1.32 | 100 | -2.672 | -0.031 | -0.455 | -2.044 | YES | YES | YES | NO | NO | NO | 0.121 | YES | YES |
| 85 | -3.59 | 1.54 | 97.175 | -2.798 | 1.278 | 0.283 | -1.188 | YES | NO | NO | YES | NO | NO | 1.102 | YES | YES |
| 86 | -3.08 | 1.659 | 92.356 | -2.895 | 1.257 | 0.485 | -1.683 | YES | NO | NO | YES | NO | NO | 1.017 | NO | NO |
| 87 | -4.02 | 1.32 | 100 | -2.672 | -0.031 | -0.336 | -2.044 | YES | YES | YES | NO | YES | NO | 0.34 | YES | YES |
| 88 | -2.14 | 1.243 | 87.898 | -3.063 | -0.422 | -0.316 | -2.802 | NO | NO | NO | NO | NO | NO | 0.551 | YES | YES |
| 89 | -3.81 | 1.352 | 99.043 | -2.466 | 0 | 0.056 | -1.539 | YES | YES | NO | NO | NO | YES | 0.226 | YES | YES |
| 90 | -2.14 | 1.229 | 94.688 | -3.069 | 0.886 | -0.045 | -2.963 | NO | NO | NO | YES | NO | NO | 1.079 | NO | YES |
| 91 | -3.84 | 1.288 | 97.184 | -2.637 | 0.562 | 0.064 | -2.118 | YES | NO | NO | NO | YES | NO | 1.198 | NO | YES |
| 92 | -4.41 | 1.353 | 92.777 | -3.625 | 0.117 | 0.003 | -1.827 | NO | NO | NO | NO | NO | NO | -0.091 | NO | YES |
| 93 | -4.47 | 0.92 | 92.429 | -3.407 | 0.119 | -0.021 | -1.917 | NO | NO | NO | NO | NO | NO | -0.114 | NO | YES |
| 94 | -4.40 | 1.319 | 94.537 | -3.67 | 0.563 | -0.105 | -1.633 | NO | NO | NO | NO | NO | YES | 0.256 | NO | YES |
| 95 | -3.84 | 1.035 | 91.668 | -2.771 | 0.055 | -0.922 | -2.891 | NO | NO | YES | NO | NO | NO | 0.216 | NO | NO |
| 96 | -2.90 | -0.052 | 84.166 | -2.735 | 0.38 | -0.959 | -2.88 | NO | NO | NO | NO | YES | NO | 1.208 | YES | NO |
| 97 | -2.91 | 0.437 | 92.372 | -2.735 | 0.285 | -1.15 | -2.841 | NO | YES | YES | NO | YES | NO | 1.14 | YES | NO |
| 98 | -2.94 | 0.491 | 93.298 | -2.735 | 0.266 | -1.016 | -2.838 | NO | NO | YES | NO | YES | NO | 1.248 | YES | NO |
| 99 | -2.93 | 0.495 | 96.401 | -2.735 | 0.237 | -1.21 | -2.788 | NO | YES | YES | NO | YES | NO | 1.164 | YES | NO |
| 100 | -2.89 | 0.129 | 87.508 | -2.735 | -1.062 | -1.409 | -2.817 | NO | NO | NO | NO | NO | NO | 1.165 | NO | NO |
| 101 | -3.24 | 1.272 | 91.97 | -2.736 | 1.808 | -0.622 | -2.014 | NO | NO | NO | NO | YES | NO | 0.875 | NO | NO |
| 102 | -2.95 | 1.463 | 95.285 | -2.632 | 0.827 | 0.369 | -3.37 | NO | NO | NO | NO | NO | NO | 0.75 | NO | NO |
| 103 | -3.17 | 0.863 | 77.971 | -2.735 | 0.738 | -1.066 | -4.007 | NO | NO | NO | NO | NO | NO | -0.771 | NO | YES |
| 104 | -4.48 | 1.635 | 98.253 | -2.532 | 0.195 | 0.095 | -1.698 | YES | YES | YES | NO | NO | YES | 0.496 | YES | NO |
| 105 | -4.06 | 1.851 | 93.829 | -2.901 | 0.229 | 0.037 | -1.52 | YES | YES | NO | NO | NO | YES | 0.349 | NO | YES |
| 106 | -3.37 | 1.832 | 94.798 | -2.895 | 0.336 | 0.296 | -2.792 | NO | YES | NO | NO | NO | NO | 0.524 | NO | YES |
| 107 | -2.29 | 1.815 | 94.747 | -3.002 | 0.986 | 0.154 | -2.847 | NO | NO | NO | NO | NO | NO | 0.844 | NO | YES |
| 108 | -2.02 | 1.833 | 93.506 | -2.788 | 0.56 | 0.301 | -2.792 | NO | NO | NO | NO | NO | NO | 0.913 | NO | YES |
| 109 | -2.12 | 1.833 | 93.03 | -2.773 | 0.569 | 0.303 | -2.792 | NO | NO | NO | YES | NO | NO | 0.965 | NO | YES |
| 110 | -2.29 | 1.815 | 94.747 | -3.002 | 0.986 | 0.154 | -2.847 | NO | NO | NO | NO | NO | NO | 0.844 | NO | YES |
| 111 | -3.40 | 0.741 | 97.379 | -2.517 | 0.942 | 0.13 | -1.532 | YES | NO | NO | YES | NO | YES | 1.247 | NO | YES |
| 112 | -4.02 | 1.32 | 100 | -2.672 | -0.031 | -0.336 | -2.044 | YES | YES | YES | NO | YES | NO | 0.34 | YES | YES |
| 113 | -3.8 | 1.682 | 97.074 | -2.826 | 1.245 | 0.318 | -1.224 | YES | NO | NO | YES | NO | YES | 1.005 | YES | YES |
| 114 | -3.35 | 1.214 | 92.303 | -2.772 | 1.357 | 0.351 | -2.044 | YES | NO | NO | YES | NO | NO | 1.02 | NO | NO |
| 115 | -1.97 | 1.17 | 86.787 | -3.057 | 0.031 | -0.23 | -2.883 | NO | NO | NO | NO | NO | NO | 0.463 | NO | YES |
| 116 | -3.07 | 0.6 | 91.796 | -2.735 | -0.792 | -0.913 | -2.603 | NO | YES | NO | NO | NO | YES | 0.718 | YES | NO |
| 117 | -3.31 | 1.211 | 91.678 | -2.826 | 1.196 | 0.238 | -2.186 | NO | YES | NO | YES | NO | NO | 1.052 | NO | YES |
| 118 | -3.37 | 0.53 | 92.912 | -2.735 | -0.715 | -1.007 | -2.416 | YES | NO | NO | NO | NO | NO | 0.716 | YES | NO |
| 119 | -3.48 | 0.918 | 92.241 | -2.745 | 0.978 | -0.322 | -2.354 | YES | NO | NO | YES | NO | NO | 0.92 | NO | YES |
| 120 | -4.11 | 1.051 | 91.775 | -2.797 | -0.061 | -0.748 | -3.083 | NO | YES | YES | NO | YES | NO | 0.531 | NO | NO |
| 121 | -3.18 | 1.265 | 94.777 | -2.615 | 0.939 | 0.065 | -2.107 | YES | NO | NO | YES | NO | NO | 1.245 | NO | YES |
| 122 | -3.22 | 1.259 | 94.551 | -2.635 | 0.934 | 0.35 | -2.096 | YES | NO | NO | YES | NO | NO | 1.226 | NO | YES |
| 123 | -3.57 | 1.215 | 94.651 | -2.744 | 1.064 | 0.084 | -2.096 | YES | NO | NO | YES | NO | NO | 1.226 | NO | YES |
| 124 | -3.44 | 1.248 | 94.549 | -2.689 | 0.995 | 0.046 | -2.088 | YES | NO | NO | YES | YES | NO | 1.214 | NO | YES |
| 125 | -2.53 | 1.232 | 96.543 | -2.832 | 0.978 | 0.162 | -2.068 | YES | NO | NO | NO | NO | YES | 1.183 | YES | NO |
| 126 | -3.40 | 0.741 | 97.379 | -2.517 | 0.942 | 0.13 | -1.532 | YES | NO | NO | YES | NO | YES | 1.247 | NO | YES |
| 127 | -4.28 | 1.226 | 95.791 | -2.918 | 0.117 | -0.584 | -2.301 | NO | YES | YES | NO | YES | NO | 0.419 | NO | NO |
| 128 | -4.36 | 1.143 | 97.474 | -2.788 | 0.071 | -0.156 | -2.144 | YES | YES | YES | NO | YES | NO | 0.412 | YES | NO |
| 129 | -3.50 | 1.03 | 92.892 | -2.897 | 0.877 | 0.482 | -1.405 | NO | YES | NO | YES | NO | YES | 1.184 | NO | YES |
| 130 | -3.83 | 1.557 | 96.966 | -2.798 | 1.35 | 0.347 | -1.163 | YES | NO | NO | YES | NO | NO | 1.023 | YES | YES |
| 131 | -3.88 | 1.316 | 100 | -2.652 | 0.033 | 0.031 | -1.641 | YES | YES | YES | NO | YES | NO | 0.107 | YES | YES |
| 132 | -3.8 | 1.682 | 97.074 | -2.826 | 1.245 | 0.318 | -1.224 | YES | NO | NO | YES | NO | YES | 1.005 | YES | YES |
| 133 | -3.23 | 1.879 | 94.08 | -2.87 | 1.172 | -0.165 | -1.779 | YES | YES | NO | YES | NO | NO | 0.932 | NO | YES |
| 134 | -4.02 | 1.32 | 100 | -2.672 | -0.031 | -0.336 | -2.044 | YES | YES | YES | NO | YES | NO | 0.34 | YES | YES |
| 135 | -4.26 | 0.623 | 96.701 | -2.684 | 0.895 | 0.118 | -1.507 | YES | NO | NO | YES | YES | NO | 1.346 | YES | NO |
| 136 | -4.34 | 0.645 | 96.705 | -2.661 | 0.962 | 0.445 | -1.445 | YES | NO | NO | YES | YES | NO | 1.34 | YES | YES |
| 137 | -3.56 | 0.697 | 91.41 | -2.702 | 1.515 | 0.163 | -1.785 | YES | YES | NO | YES | YES | NO | 1.071 | NO | NO |
| 138 | -2.00 | 1.763 | 94.185 | -2.35 | 0.093 | 0.674 | -2.109 | YES | NO | NO | NO | NO | NO | 0.222 | NO | NO |
| 139 | -3.11 | 1.662 | 93.985 | -2.275 | 0.151 | 0.203 | -1.875 | YES | NO | NO | NO | NO | NO | 0.419 | YES | NO |
| 140 | -1.09 | 1.476 | 91.311 | -2.731 | 1.098 | -0.259 | -2.053 | NO | NO | NO | NO | NO | NO | 0.868 | NO | YES |
| 141 | -3.09 | 0.686 | 94.187 | -2.735 | -0.359 | -0.584 | -2.473 | NO | NO | NO | NO | NO | NO | 1.035 | YES | NO |
| 142 | -3.44 | 0.997 | 91.603 | -3.012 | 1.072 | 0.337 | -2.102 | YES | NO | NO | YES | NO | YES | 1.162 | NO | NO |
| 143 | -3.15 | 1.121 | 91.319 | -2.886 | 1.266 | 0.005 | -2.214 | YES | NO | NO | YES | NO | NO | 1.058 | NO | NO |
| 144 | -3.35 | 1.291 | 95.043 | -3.157 | 0.609 | -0.75 | -2.917 | NO | YES | NO | NO | YES | YES | 0.948 | NO | NO |
| 145 | -3.10 | 1.245 | 96.108 | -2.967 | 0.727 | -0.31 | -2.287 | YES | NO | NO | NO | NO | NO | 1.042 | NO | NO |
| 146 | -3.42 | 1.148 | 93.194 | -2.769 | 1.467 | 0.171 | -1.979 | YES | NO | NO | YES | NO | NO | 1.032 | NO | NO |
| 147 | -3.59 | 1.091 | 92.597 | -2.766 | 2.049 | 0.257 | -2.238 | YES | NO | NO | NO | NO | NO | 1.29 | NO | YES |
| 148 | -2.66 | 0.621 | 75.756 | -2.736 | 0.092 | -0.316 | -3.171 | NO | NO | NO | NO | NO | NO | 0.511 | NO | YES |
| 149 | -3.52 | 1.06 | 97.425 | -3.017 | 0.872 | 0.28 | -3.076 | NO | NO | NO | NO | NO | NO | 1.155 | NO | YES |
| 150 | -1.25 | 1.131 | 70.955 | -2.735 | -0.494 | -0.26 | -3.433 | NO | NO | NO | NO | NO | NO | 0.465 | NO | NO |
| 151 | -2.57 | 1.827 | 96.538 | -3.372 | 0.749 | 0.316 | -3.394 | NO | NO | NO | NO | NO | NO | 1.38 | NO | NO |
| 152 | -2.57 | 1.827 | 96.538 | -3.372 | 0.749 | 0.316 | -3.394 | NO | NO | NO | NO | NO | NO | 1.38 | NO | NO |
| 153 | -1.74 | 1.575 | 100 | -3.252 | 0.72 | -0.025 | -3.405 | NO | NO | NO | NO | NO | NO | 1.112 | NO | YES |
| 154 | -2.38 | 1.169 | 85.108 | -3.924 | 0.691 | -0.214 | -3.078 | NO | NO | NO | NO | NO | NO | 1.271 | NO | NO |
| 155 | -3.52 | 1.06 | 97.425 | -3.017 | 0.872 | 0.284 | -3.076 | NO | NO | NO | NO | NO | NO | 1.155 | NO | YES |
| 156 | -3.52 | 1.06 | 97.425 | -3.017 | 0.872 | 0.284 | -3.076 | NO | NO | NO | NO | NO | NO | 1.155 | NO | YES |
| 157 | -3.40 | 0.956 | 95.281 | -3.127 | 0.587 | -0.433 | -3.13 | NO | NO | NO | NO | NO | NO | 1.399 | NO | YES |
| 158 | -3.57 | 1.024 | 98.006 | -2.738 | 0.447+ | -0.643 | -3.124 | NO | NO | NO | NO | NO | NO | 0.93 | NO | YES |
| 159 | -3.20 | 0.896 | 97.008 | -2.738 | 0.117 | -0.564 | -3.193 | NO | NO | NO | NO | NO | NO | 0.979 | NO | YES |
| 160 | -3.69 | 0.965 | 100 | -2.736 | 0.365 | -0.75 | -3.197 | NO | NO | NO | NO | NO | NO | 0.965 | NO | YES |
| 161 | -2.46 | 0.297 | 66.142 | -2.735 | 1.198 | -1.19 | -3.201 | NO | NO | NO | NO | NO | NO | 0.384 | NO | NO |
| 162 | -3.68 | 1.072 | 95.859 | -3.204 | 0.832 | 0.322 | -3.089 | NO | NO | NO | NO | NO | NO | 1.191 | NO | YES |
| 163 | -2.03 | 1.578 | 96.859 | -3.204 | 0.745 | -0.007 | -3.285 | NO | NO | NO | NO | NO | NO | 1.098 | NO | YES |
| 164 | -2.11 | 1.787 | 98.515 | -2.918 | 0.074 | 0.186 | -3.285 | NO | NO | NO | NO | NO | NO | 0.757 | NO | NO |
| 165 | -1.25 | 1.131 | 70.955 | -2.735 | -0.494 | -0.26 | -3.433 | NO | NO | NO | NO | NO | NO | 0.465 | NO | NO |
| 166 | -2.09 | 1.705 | 100 | -3.516 | 0.645 | -0.075 | -3.603 | NO | NO | NO | NO | NO | NO | 1.122 | NO | NO |
| 167 | -2.09 | 1.705 | 100 | -3.156 | 0.645 | -0.075 | -3.603 | NO | NO | NO | NO | NO | NO | 1.122 | NO | NO |
| 168 | -0.64 | 1.161 | 94.453 | -4.028 | 0.054 | -0.241 | -3.078 | NO | NO | NO | NO | NO | NO | 0.682 | NO | YES |
| 169 | -3.23 | 1.204 | 94.064 | -3.126 | 0.771 | -0.144 | -2.224 | NO | NO | NO | NO | NO | NO | 0.912 | NO | NO |
| 170 | -4.69 | 0.993 | 97.664 | -2.634 | 0.21 | -0.689 | -2.101 | YES | YES | YES | NO | YES | NO | 0.162 | YES | NO |
| 171 | -2.15 | 1.945 | 94.537 | -2.937 | 0.827 | 0.207 | -3.002 | NO | NO | NO | YES | NO | NO | 0.992 | NO | YES |
| 172 | -4.90 | 1.977 | 97.092 | -2.573 | 0.365 | 0.003 | -1.518 | YES | YES | YES | NO | NO | NO | 0.175 | YES | NO |
| 173 | -3.31 | 1.875 | 91.057 | -2.802 | 1.158 | 0.262 | -1.816 | YES | NO | NO | YES | NO | NO | 0.952 | NO | YES |
| 174 | -3.33 | 1.79 | 91.906 | -2.682 | 1.264 | 0.544 | -1.513 | YES | YES | NO | YES | NO | NO | 0.975 | YES | YES |
| 175 | -3.28 | 2.034 | 92.01 | -2.802 | 0.978 | 0.077 | -1.637 | YES | YES | NO | YES | NO | NO | 1.026 | NO | YES |
| 176 | -3.29 | 1.915 | 92.014 | -2.752 | 1.124 | 0.481 | -1.575 | YES | YES | NO | YES | NO | NO | 0.992 | YES | YES |
| 177 | -3.37 | 0.897 | 90.999 | -2.795 | 1.033 | -0.137 | -2.134 | NO | NO | NO | YES | NO | NO | 0.972 | NO | YES |
| 178 | -3.32 | 1.222 | 90.859 | -2.811 | 1.047 | 0.004 | -2.042 | YES | NO | NO | YES | NO | NO | 1.003 | NO | YES |
| 179 | -3.33 | 1.79 | 91.906 | -2.682 | 1.264 | 0.544 | -1.513 | YES | YES | NO | YES | NO | NO | 0.975 | YES | YES |
| 180 | -4.34 | 1.379 | 91.948 | -2.588 | 0.396 | 0.223 | -2.862 | NO | NO | NO | NO | NO | NO | 0.744 | NO | NO |
| 181 | -3.51 | 1.118 | 95.421 | -3.159 | -0.162 | 0.149 | -3.209 | NO | NO | NO | NO | NO | NO | 0.705 | NO | NO |
| 182 | -1.97 | 0.528 | 84.181 | -2.81 | 0.337 | -0.429 | -2.937 | NO | NO | NO | NO | NO | NO | 0.425 | NO | YES |
| 183 | -4.28 | 0.427 | 76.223 | -2.735 | 0.439 | -1.081 | -3.64 | NO | NO | NO | NO | NO | NO | 0.321 | NO | YES |
| 184 | -3.83 | 0.573 | 86.045 | -3.004 | -0.213 | -0.947 | -2.219 | YES | YES | YES | NO | YES | NO | 0.26 | NO | NO |
| 185 | -3.33 | 1.038 | 89.381 | -2.984 | 0.017 | -0.818 | -2.066 | YES | YES | YES | NO | YES | NO | 0.267 | YES | NO |
| 186 | -4.67 | 1.064 | 92.723 | -2.848 | 0.631 | -0.778 | -2 | NO | YES | YES | NO | YES | NO | 0.696 | YES | YES |
| 188 | -3.98 | 0.955 | 93.637 | -2.859 | 0.471 | -0.734 | -2.223 | YES | YES | NO | NO | NO | NO | 0.516 | YES | NO |
| 189 | -5.28 | 0.926 | 92.828 | -2.829 | 0.819 | -0.431 | -2.018 | NO | YES | YES | NO | YES | NO | 0.619 | NO | YES |
| 190 | -2.67 | 1.473 | 49.241 | -2.736 | 1.442 | -0.894 | -3.439 | NO | NO | NO | NO | NO | NO | 1.481 | NO | YES |
| 191 | -2.63 | 1.405 | 50.208 | -2.736 | 1.416 | -0.953 | -3.454 | NO | NO | NO | NO | NO | NO | 1.522 | NO | YES |
| 192 | -3.54 | 1.426 | 58.75 | -2.781 | 0.363 | -1.059 | -3.256 | NO | NO | NO | NO | NO | NO | 0.342 | NO | YES |
| 193 | -1.89 | 0.268 | 80.914 | -3.019 | 0.408 | -0.719 | -3.808 | NO | NO | NO | NO | NO | NO | 0.971 | NO | YES |
| 194 | -5.34 | 1.017 | 100 | -2.762 | 0.356 | -0.788 | -3.364 | NO | NO | NO | NO | YES | NO | 0.522 | NO | NO |
| 195 | -3.63 | 0.986 | 93.735 | -2.832 | 1.278 | -0.014 | -2.167 | NO | NO | NO | YES | NO | YES | 1.077 | YES | NO |
| 196 | -3.03 | 1.11 | 93.039 | -2.849 | 1.404 | -0.03 | -2.274 | NO | NO | NO | YES | NO | NO | 1.027 | NO | NO |
| 197 | -3.61 | 1.001 | 93.515 | -2.823 | 1.41 | 0.353 | -1.786 | NO | NO | NO | YES | NO | YES | 1.061 | YES | YES |
| 198 | -3.95 | 0.638 | 100 | -2.736 | -0.254 | -1.646 | -4.218 | NO | NO | NO | NO | NO | NO | -0.116 | NO | YES |
| 199 | -3.00 | 0.926 | 79.792 | -2.762 | -0.318 | -0.721 | -3.932 | NO | NO | NO | NO | NO | NO | 0.358 | NO | YES |
| 200 | -2.99 | 0.835 | 73.859 | -3.041 | -3.041 | -0.343 | -3.645 | NO | NO | NO | NO | NO | NO | 0.196 | NO | NO |
| 201 | -4.50 | 0.808 | 86.193 | -3.09 | -0.349 | -0.771 | -3.378 | NO | NO | NO | NO | NO | NO | 0.93 | NO | YES |
| 202 | -3.68 | 0.73 | 80.132 | -2.853 | 0.203 | -0.788 | -3.376 | NO | NO | NO | NO | NO | NO | 0.812 | NO | YES |
| 203 | -4.07 | 0.841 | 79.024 | -3.229 | -0.226 | -1.007 | -3.372 | NO | NO | NO | NO | NO | NO | 0.879 | NO | YES |
| 204 | -4.02 | 0.814 | 81.974 | -2.96 | -0.007 | -1.044 | -3.372 | NO | NO | NO | NO | NO | NO | 0.858 | YES | YES |
| 205 | -3.41 | 0.515 | 96.354 | -2.735 | -0.428 | 0.05 | -2.571 | NO | NO | NO | NO | NO | NO | 0.872 | YES | NO |
| 206 | -1.23 | 0.836 | 86.496 | -3.572 | 0.419 | -0.508 | -3.086 | NO | NO | NO | NO | NO | NO | 1.069 | NO | YES |
| 207 | -1.62 | 1.194 | 93.165 | -3.43 | 0.506 | -0.07 | -3.033 | NO | NO | NO | NO | NO | NO | 0.839 | NO | YES |
| 208 | 0.25 | 1.565 | 94.872 | -3.289 | 0.317 | -0.087 | -3.43 | NO | NO | NO | NO | NO | NO | 1.37 | NO | NO |
| 209 | -1.16 | 0.622 | 68.393 | -2.735 | -0.546 | -0.897 | -3.161 | NO | NO | NO | NO | NO | NO | 0.086 | NO | YES |
| 210 | -3.08 | 0.951 | 80.588 | -2.751 | 0.927 | -0.475 | -2.108 | NO | NO | NO | NO | NO | NO | 0.187 | NO | YES |
| 211 | -2.92 | 0.116 | 88.298 | -2.735 | -0.888 | 0.272 | -0.444 | NO | NO | NO | NO | NO | NO | -0.669 | NO | NO |
| 212 | -3.03 | 0.583 | 87.08 | -2.735 | 0.138 | 0.032 | -2.585 | NO | YES | NO | NO | YES | NO | 1.067 | YES | NO |
| 213 | -3.17 | 0.699 | 89.907 | -2.735 | 0.329 | -0.334 | -2.53 | NO | NO | NO | NO | YES | NO | 1.135 | YES | NO |
| 214 | -3.24 | 1.871 | 93.762 | -2.881 | 1.198 | 0.511 | -1.748 | YES | YES | NO | YES | NO | NO | 1.025 | NO | YES |
| 215 | -3.26 | 0.761 | 90.004 | -2.735 | 0.323 | -0.403 | -2.577 | NO | NO | NO | NO | YES | NO | 1.148 | YES | NO |
| 216 | -4.61 | 0.398 | 97.949 | -2.735 | -0.816 | -1.554 | -2.439 | NO | NO | NO | NO | NO | NO | 0.121 | NO | NO |
| 217 | -3.33 | 0.721 | 97.907 | -2.735 | -0.145 | -1.307 | -2.324 | NO | YES | NO | NO | YES | NO | 1.54 | NO | NO |
| 218 | -2.99 | 0.152 | 87.969 | -2.735 | 0.415 | -1.413 | -2.641 | NO | NO | NO | NO | NO | NO | 1.342 | YES | NO |
| 219 | -2.31 | 1.436 | 94.425 | -3.329 | 0.851 | 0.381 | -3.343 | NO | NO | NO | NO | NO | YES | 0.659 | NO | NO |
| 220 | -2.41 | 1.435 | 93.949 | -3.302 | 0.861 | 0.403 | -3.343 | NO | NO | NO | NO | NO | YES | 0.744 | NO | NO |
| 221 | -5.03 | 1.884 | 97.916 | -2.592 | 0.461 | -0.116 | -1.525 | YES | YES | YES | NO | NO | NO | 0.202 | YES | YES |
| 222 | -3.20 | 1.759 | 94.187 | -2.777 | 1.304 | 0.484 | -1.733 | YES | YES | NO | YES | NO | NO | 0.954 | NO | YES |
| 223 | -3.81 | 1.813 | 97.079 | -2.845 | 1.14 | -0.314 | -1.271 | NO | NO | NO | NO | NO | YES | 1.063 | YES | YES |
| 224 | -3.81 | 1.803 | 96.864 | -2.851 | 1.152 | 0.123 | -1.255 | YES | NO | NO | YES | NO | NO | 1.04 | YES | YES |
| 225 | -3.20 | 1.175 | 95.484 | -3.255 | 0.569 | -0.532 | -1.996 | YES | YES | NO | YES | NO | YES | 0.897 | YES | NO |
| 226 | -5.27 | 1.372 | 97.148 | -2.715 | -0.204 | 0.45 | -1.696 | YES | YES | YES | NO | NO | NO | 0.07 | YES | YES |
| 227 | -5.15 | 1.822 | 98.178 | -2.604 | 0.238 | 0.038 | -1.41 | YES | YES | YES | NO | YES | NO | 0.111 | YES | NO |
| 228 | -5.07 | 1.051 | 97.772 | -2.657 | 0.058 | -0.288 | -1.784 | YES | YES | YES | NO | YES | NO | 0.059 | YES | NO |
| 229 | -5.38 | 1.316 | 99.104 | -2.701 | -0.193 | 0.009 | -1.843 | YES | YES | YES | NO | YES | NO | 0.088 | YES | NO |
| 230 | -2.41 | 1.812 | 92.367 | -2.716 | 0.956 | 0.548 | -2.559 | YES | NO | NO | YES | NO | NO | 0.829 | NO | YES |
| 231 | -2.76 | 1.044 | 92.301 | -2.793 | 0.972 | 0.291 | -2.501 | YES | NO | NO | YES | NO | NO | 0.661 | NO | YES |
| 232 | -2.90 | 1.222 | 91.212 | -2.769 | 1.127 | 0.194 | -2.483 | NO | NO | NO | YES | NO | NO | 0.858 | NO | YES |
| 233 | -3.02 | 0.989 | 89.935 | -3.155 | 0.81 | 0.349 | -1.888 | NO | NO | NO | YES | NO | NO | 0.638 | NO | YES |
| 234 | -2.31 | 0.634 | 72.66 | -3.319 | 0.321 | -0.27 | -2.968 | NO | NO | NO | NO | NO | NO | 0.84 | NO | YES |
| 235 | -2.31 | 0.514 | 60.09 | -3.302 | 0.374 | -0.506 | -3.017 | NO | NO | NO | NO | NO | NO | 0.841 | NO | YES |
| 236 | -3.25 | 1.656 | 92.362 | -3 | 1.436 | 0.583 | -2.365 | NO | NO | NO | YES | NO | NO | 0.886 | YES | NO |
| 237 | -2.90 | 1.101 | 94.237 | -2.974 | 1.372 | 0.341 | -2.591 | NO | NO | NO | YES | NO | YES | 0.686 | NO | YES |
| 238 | -3.03 | 1.065 | 94.565 | -3.189 | 0.966 | -0.275 | -2.296 | NO | NO | NO | YES | NO | YES | 0.724 | NO | YES |
| 239 | -3.97 | 1.161 | 98.533 | -3.113 | 1.126 | 0.009 | -2.262 | NO | NO | NO | NO | NO | NO | 0.8 | NO | NO |
| 240 | -3.11 | 1.08 | 95.197 | -2.947 | 1.11 | 0.291 | -2.397 | NO | NO | NO | YES | NO | YES | 0.685 | YES | YES |
| 241 | -3.40 | 1.044 | 95.92 | -3.26 | 0.93 | 0.503 | -2.388 | NO | NO | NO | YES | NO | NO | 0.802 | NO | YES |
| 242 | -2.98 | 1.082 | 94.027 | -3.127 | 1.226 | 0.53 | -2.743 | NO | NO | NO | YES | NO | YES | 0.708 | NO | YES |
| 243 | -3.76 | 1.183 | 98.194 | -3.356 | 0.95 | -0.006 | -2.203 | NO | NO | NO | YES | NO | YES | 0.574 | NO | YES |
| 244 | -1.94 | 0.844 | 71.555 | -2.845 | 0.828 | -0.064 | -2.768 | NO | NO | NO | NO | NO | YES | 1.121 | NO | YES |
| 245 | -3.02 | 0.563 | 87.886 | -2.735 | 1.273 | 0.534 | -0.145 | YES | YES | NO | YES | NO | YES | 1.103 | NO | YES |
| 246 | -2.93 | 1.034 | 94.044 | -2.736 | 0.538 | 0.169 | -2.101 | NO | NO | NO | NO | NO | NO | 0.335 | NO | NO |
| 247 | -2.713 | 1.459 | 92.857 | -2.893 | 0.526 | -0.053 | -3.657 | NO | NO | NO | NO | NO | NO | 0.932 | NO | YES |
| 248 | -3.015 | 0.603 | 76.82 | -3.962 | 0.501 | -0.421 | -3.123 | NO | NO | NO | NO | NO | NO | 0.63 | NO | YES |
| 249 | -3.04 | 1.195 | 78.855 | -3.776 | 0.531 | -0.075 | -3.141 | NO | NO | NO | NO | NO | NO | 0.915 | NO | YES |
| 250 | -3.077 | 0.599 | 76.828 | -3.958 | 0.51 | -0.453 | -3.118 | NO | NO | NO | NO | NO | NO | 0.714 | NO | YES |
| 251 | -3.114 | 1.207 | 78.863 | -3.763 | 0.542 | -0.08 | -3.135 | NO | NO | NO | NO | NO | NO | 0.999 | NO | YES |
| 252 | -3.472 | 0.444 | 87.099 | -3.822 | 0.246 | -0.198 | -3.021 | NO | NO | NO | NO | NO | NO | 1.064 | NO | NO |
| 253 | -2.363 | 1.557 | 94.479 | -3.46 | 0.729 | 0.004 | -3.51 | NO | NO | NO | NO | NO | NO | 0.735 | NO | NO |
| 254 | -3.636 | 0.6 | 76.961 | -4.117 | 0.225 | -0.473 | -3.111 | NO | NO | NO | NO | NO | NO | 1.082 | NO | NO |
| 255 | -2.892 | 1.126 | 79.839 | -3.313 | 0.648 | -0.192 | -3.141 | NO | NO | NO | NO | NO | NO | 0.991 | NO | NO |
| 256 | -2.937 | 1.143 | 79.602 | -3.295 | 0.668 | -0.163 | -3.141 | NO | NO | NO | NO | NO | NO | 1.004 | NO | NO |
| 257 | -2.579 | 1.162 | 79.126 | -3.751 | 0.683 | -0.093 | -3.83 | NO | NO | NO | NO | NO | NO | 0.752 | NO | YES |
| 258 | -2.658 | 1.174 | 79.133 | -3.734 | 0.693 | -0.098 | -3.83 | NO | NO | NO | NO | NO | NO | 0.837 | NO | YES |
| 259 | -2.36 | 1.189 | 77.494 | -3.722 | 0.674 | -0.082 | -3.594 | NO | NO | NO | NO | NO | NO | 0.687 | NO | YES |
| 260 | -2.852 | 0.346 | 62.367 | -3.771 | 0.363 | -0.195 | -3.512 | NO | NO | NO | NO | NO | NO | 0.794 | NO | NO |
| 261 | -3.309 | 1.436 | 93.434 | -3.11 | 0.554 | 0.407 | -3.461 | NO | NO | NO | NO | NO | NO | 1.106 | NO | NO |
| 262 | -3.309 | 1.193 | 79.266 | -3.8 | 0.399 | -0.104 | -3.948 | NO | NO | NO | NO | NO | NO | 1.093 | NO | NO |
| 263 | -3.309 | 1.436 | 93.434 | -3.11 | 0.554 | 0.407 | -3.461 | NO | NO | NO | NO | NO | NO | 1.096 | NO | NO |
| 264 | -0.858 | 1.406 | 99.64 | -3.103 | 0.752 | -0.149 | -3.118 | NO | NO | NO | NO | NO | NO | 0.796 | NO | YES |
| 265 | -1.159 | 1.423 | 100 | -2.981 | 0.808 | -0.21 | -3.153 | NO | NO | NO | NO | NO | NO | 0.64 | NO | YES |
| 266 | -2.162 | 1.528 | 100 | -3.344 | 0.139 | -0.173 | -3.3 | NO | NO | NO | NO | NO | NO | 0.5 | NO | YES |
| 267 | -2.561 | 1.537 | 100 | -3.28 | 0.142 | -0.168 | -3.293 | NO | NO | NO | NO | NO | NO | 0.567 | NO | YES |
| 268 | -2.15 | 1.185 | 93.914 | -3.022 | 0.728 | -0.157 | -3.267 | NO | NO | NO | NO | NO | NO | 0.93 | NO | YES |
| 269 | -3.43 | 1.185 | 92.043 | -2.754 | 1.218 | 0.095 | -2.096 | YES | NO | NO | YES | NO | NO | 0.789 | NO | NO |
| 270 | -1.91 | 1.807 | 91.896 | -2.947 | 0.685 | -0.092 | -2.957 | NO | NO | NO | YES | NO | NO | 1.078 | NO | NO |
| 271 | -4.00 | 1.62 | 100 | -2.458 | 0.205 | 0.309 | -1.554 | YES | YES | NO | NO | NO | NO | 0.244 | YES | NO |
| 272 | -2.27 | 1.75 | 93.571 | -3.032 | 0.891 | 0.498 | -2.957 | NO | NO | NO | NO | NO | NO | 0.951 | NO | YES |
| 273 | -3.58 | 1.246 | 96.189 | -2.894 | 0.575 | -0.37 | -2.191 | YES | NO | NO | NO | NO | YES | 1.028 | YES | NO |
| 274 | -4.05 | 0.673 | 96.216 | -2.71 | 0.645 | 0.0041 | -1.36 | YES | YES | NO | YES | NO | NO | 1.004 | YES | NO |
| 275 | -5.23 | 2.017 | 99.152 | -2.702 | 0.385 | 0.107 | -1.442 | YES | NO | NO | YES | YES | NO | 1.056 | YES | NO |
| 276 | -2.98 | 1.846 | 97.684 | -2.577 | 0.861 | 0.61 | -1.59 | YES | NO | NO | YES | NO | YES | 1.282 | NO | YES |
| 277 | -3.64 | 1.174 | 97.301 | -3.157 | 0.738 | -0.082 | -2.13 | NO | YES | NO | NO | NO | YES | 0.882- | NO | NO |
| 278 | -1.73 | 1.127 | 98.007 | -2.736 | 0.289 | -0.265 | -2.977 | NO | NO | NO | NO | NO | NO | 0.195 | NO | YES |
| 279 | -1.16 | 1.704 | 89.839 | -2.751 | 0.687 | 0.179 | -2.537 | NO | NO | NO | NO | NO | NO | 0.966 | NO | NO |
| 280 | -3.46 | 1.59 | 94.444 | -3.131 | 0.158 | 0.176 | -1.879 | NO | YES | NO | NO | NO | YES | 0.236 | NO | NO |

| **Table S4** Prediction of toxicity of secondary metabolites inhibiting metabolic enzymes using ProTox-II | | | | |
| --- | --- | --- | --- | --- |
| **Alkaloids** | **LD50 (mg/kg)** | **Toxicity class** | **Active target** | **Probability** |
| Aconitine | 1 | 1 | Immunotoxicity | 0.99 |
| Allocryptopine | 940 | 4 | Immunotoxicity | 0.97 |
| Alopecurin A | 5000 | 5 | - | - |
| Aloperine | 480 | 4 | - | - |
| Alstofolinine A | 3000 | 5 | Immunotoxicity | 0.93 |
| Alstonoxine C | 325 | 4 | – | – |
| Alstonoxine D | 325 | 4 | Immunotoxicity | 0.92 |
| Anolobine | 450 | 4 | Immunotoxicity | 0.96 |
| Anonaine | 450 | 4 | Immunotoxicity | 0.76 |
| Arecaine | 1000 | 4 | - | - |
| Arecoline | 550 | 4 | Mutagenicity | 0.71 |
| Aromoline | 1700 | 4 | Immunotoxicity  Mutagenicity | 0.99  0.71 |
| Asimilobine | 240 | 3 | - | - |
| Atropine | 380 | 4 | – | – |
| Augustamine | 765 | 4 | Immunotoxicity | 0.94 |
| Berbamine | 1700 | 4 | Immunotoxicity  Mutagenicity | 0.99  0.77 |
| Berberine | 200 | 3 | Immunotoxicity  Cytotoxicity  Aryl hydrocarbon Receptor (AhR)  Mitochondrial Membrane Potential (MMP) | 0.99  0.96  0.87  0.87 |
| Berbidine | 1700 | 4 | Immunotoxicity | 0.90 |
| Berbostrejdine | 1180 | 4 | Immunotoxicity | 0.98 |
| Bergenin | 765 | 4 | Immunotoxicity | 0.99 |
| Bersavine | 1700 | 4 | Immunotoxicity | 0.99 |
| Bicuculine | 1000 | 4 | Immunotoxicity  Aryl hydrogen receptor | 0.97  0.76 |
| Bubbialidine | 2573 | 5 | - | - |
| Cabucraline | 406 | 4 | – | – |
| Caffeine | 127 | 3 | - | - |
| Camptothecin | 50 | 3 | Immunotoxicity | 0.99 |
| Cathachunine | 68 | 2 | Immunotoxicity  Cytotoxicity  ATPase family AAA domain containing protein (ATAD5) | 0.99  0.82  0.82 |
| Cepharanoline | 1900 | 4 | Immunotoxicity  Mutagenicity | 0.99  0.76 |
| Cepharanthine | 1900 | 4 | Immunotoxicity  Mutagenicity | 0.99  0.88 |
| Chelerythrine | 778 | 4 | Immunotoxicity  Mutagenicity  Aryl hydrogen Receptor  Nuclear factor (erythroid-derived 2)-like 2/ antioxidant responsive elements (nrf2/ARE)  Heat shock factor response element (HSE) | 0.99  0.77  0.94  0.85  0.85 |
| Chelidonine | 460 | 4 | Immunotoxicity | 0.95 |
| Cherianoine | 1000 | 4 | Immunotoxicity | 0.87 |
| Choline | 1391 | 4 | -- | -- |
| Chreylline | 500 | 4 | Immunotoxicity | 0.75 |
| Coclaurine | 2000 | 4 | Immunotoxicity | 0.85 |
| codeine | 85 | 3 | Androgen Receptor | 1 |
| Columbamine | 200 | 3 | Immunotoxicity  Mitochondrial membrane potential (MMP) | 0.98  0.78 |
| Coptisine | 200 | 3 | Immunotoxicity | 0.87 |
| Corynoxidine | 928 | 4 | Immunotoxicity | 0.88 |
| Crebanine | 89470 | 6 | Immunotoxicity,  Mutagenicity | 0.99  0.74 |
| Crinine | 155 | 3 | Immunotoxicity | 0.91 |
| Cryptolepine | 2000 | 4 | - | - |
| Cryptolepinone | 300 | 3 | - | - |
| Cycleanine | 28 | 2 | Immunotoxicity  Mutagenicity | 0.99  0.84 |
| cytisine | 101 | 3 | - | - |
| D7 Mesembrenone | 400 | 4 | - | - |
| Dauricine | 1180 | 4 | Immunotoxicity | 0.97 |
| Dehassiline | 928 | 4 | Immunotoxicity | 0.80 |
| Dehydotylophorine | 500 | 4 | Mitochondrial membrane potential | 0.70 |
| Dehydroantofine | 500 | 4 | Immunotoxicity | 0.97 |
| Dehydrocorydaline | 200 | 3 | Immunotoxicity  Mitochondrial Membrane Potential(MMP) | 0.96  0.80 |
| Dehydrocrebaine | 450 | 4 | Immunotoxicity,  Mutagenicity,  Aryl hydrocarbon Receptor | 0.98  0.84  0.72 |
| Dehydronantenine | 450 | 4 | Immunotoxicity  Mutagenicity  Aryl hydrocarbon Receptor(AhR) | 0.99  0.84  0.72 |
| Dehydroroemerine | 450 | 4 | - | - |
| Dehyrodicentrine | 450 | 4 | Immunotoxicity  Mutagenicity  Aryl hydrocarbon Receptor(AhR) | 0.98  0.84  0.72 |
| Dicentrine |  |  |  |  |
| Didehydroglaucine | 900 | 4 | Immunotoxicity | 0.96 |
| Dihydrosanguinarine | 500 | 4 | Immunotoxicity | 0.92 |
| Echinulin | 400 | 4 | Immunotoxicity | 0.86 |
| Epigasine A | 300 | 3 | Immunotoxicity | 0.98 |
| Epigasine B | 2000 | 4 | Immunotoxicity | 0.98 |
| Epimesembranol | 420 | 4 | Immunotoxicity | 0.95 |
| Fangchinoline | 1700 | 4 | Immunotoxicity  Mutagenicity | 0.99  0.77 |
| Fississaine | 200 | 3 | Immunotoxicity | 0.98 |
| Flueggether A | 270 | 3 | - | - |
| Fluevirosine E | 270 | 3 | Immunotoxicity | 0.85 |
| Fluevirosine F | 75 | 3 | Immunotoxicity | 0,70 |
| Fluevirosine G | 2573 | 5 | Immunotoxicity | 0.95 |
| Fluggenine H | 270 | 3 | Immunotoxicity | 0.96 |
| Fluggenine A | 75 | 3 | - | - |
| Fluggenine B | 270 | 3 | Immunotoxicity | 0.90 |
| Fluggenine C | 75 | 3 | - | - |
| Fluggenine E | 270 | 3 | Immunotoxicity | 0.99 |
| Fluggenine G | 75 | 3 | - | - |
| Galantamine | 85 | 3 | Immunotoxicity  Aryl hydrocarbon receptor | 0.98  1.0 |
| Glaucine | 350 | 4 | Immunotoxicity | 0.99 |
| Guvacine | 1000 | 4 | - | - |
| Hamayne | 155 | 3 | Immunotoxicity | 0.74 |
| Harmaline | 550 | 4 | - | - |
| Harmine | 500 | 4 | - | - |
| Heyneanine | 40 | 2 | Immunotoxicity | 0.95 |
| Hippadine | 1000 | 4 | Immunotoxicity  Mutagenicity | 0.98  0.71 |
| Hodgkinsine | 15 | 2 | - | - |
| Hordenine | 2859 | 5 | - | - |
| Ismine | 2100 | 5 | Immunotoxicity | 0.84 |
| Isocorydine | 350 | 4 | Immunotoxicity | 0.99 |
| Jatrorrhizine | 200 | 3 | Immunotoxicity  Mitochondrial membrane potential (MMP) | 0.98  0.73 |
| Lanuginosine | 450 | 3 | Immunotoxicity  Mutagenicity  Cytotoxicity | 0.99  0.93  0.70 |
| Laurotetanine | 450 | 4 | Immunotoxicity | 0,98 |
| Linderaggrine A | 1600 | 4 | - | - |
| Liriodenine | 450 | 3 | Immunotoxicity  Mutagenicity | 0.99  0.85 |
| L-Tetrahydropalmatine | 580 | 4 | Immunotoxicity | 0.76 |
| lycoranine B | 778 | 4 | Immunotoxicity  cytotoxicity | 0.99  0.8 |
| Lycorine | 230 | 3 | Immunotoxicity  cytotoxicity | 0.87  0.75 |
| Lysicamine | 450 | 4 | Immunotoxicity  Mutagenicity | 0.99  0.85 |
| Macrocarpine D | 370 | 4 | – | – |
| Macrodasine H | 760 | 4 | Immunotoxicity | 0.97 |
| Magnoflorine | 401 | 4 | Immunotoxicity | 0.94 |
| Matrine | 243 | 3 | - | - |
| Menisdaurilide | 55 | 3 | - | - |
| Mesembranol | 420 | 4 | Immunotoxicity | 0.95 |
| Mesembrenone | 580 | 4 | Immunotoxicity | 0.94 |
| Mesembrine | 369 | 4 | Immunotoxicity | 0.73 |
| Montanine | 1250 | 4 | Mutagenicity | 0.73 |
| Morphine | 335 | 4 | - | - |
| Muraricine | 1700 | 4 | Immunotoxicity | 0.98 |
| N- methylcalycinine | 450 | 4 | Immunotoxicity | 0.99 |
| N- methyllaurotetanine | 350 | 4 | Immunotoxicity | 0.99 |
| N-acylcytisine | 511 | 4 | - | - |
| Nantenine | 401 | 4 | Immunotoxicity  Mutagenicity | 0.99  0.80 |
| Narcotine | 840 | 4 | immunotoxicity | 0.98 |
| N-chloromethyl ungiminorine | 155 | 3 | Immunotoxicity | 0.99 |
| Neochinulin A | 500 | 4 | - | - |
| Neochinulin D | 500 | 4 | Immunotoxicity | 0.76 |
| Neoline | 200 | 3 | - | - |
| N-formylcytisine | 511 | 4 | - | - |
| Nigritanine | 1000 | 4 | – | – |
| Niruroidine | 75 | 3 | ATPase family AAA domain-containing protein 5 | 0.72 |
| N-methylcytisine | 101 | 3 | - | - |
| N-methylouregidione | 2000 | 4 | Immunotoxicity | 0.71 |
| Norpharman | 260 | 3 | Aryl hydrocarbon receptor9AHR) | 1.0 |
| Norsanguinarine | 778 | 4 | Immunotoxicity | 0.92 |
| Norsecurinic acid | 75 | 3 | - | - |
| Obamegine | 1700 | 4 | Immunotoxicity  Mutagenicity | 0.99  0.71 |
| O-demethyllycoramine N-oxide | 127 | 3 | Immunotoxicity | 0.92 |
| Oleraciamid G | 500 | 4 | - | - |
| Oleracimine | 600 | 4 | - | - |
| Oleracimine A | 4000 | 5 | - | - |
| Oleracone | 825 | 4 | - | - |
| Oleraindole A | 685 | 4 | - | - |
| Oleraindole B | 685 | 4 | Immunotoxicity | 0.89 |
| Oleraindole D | 1000 | 4 | Immunotoxicity | 0.99 |
| O-methybulbocapine | 401 | 4 | Immunotoxicity  Mutagenicity | 0.99  0.80 |
| O-methylbulbocapnine | 401 | 4 | Immunotoxicity  Mutagenicity | 0.99  0.80 |
| O-methylismine | 2100 | 5 | Immunotoxicity | 0.93 |
| Ouregidione | 2000 | 4 | - | - |
| Oxostephanine | 450 | 4 | Immunotoxicity  Mutagenicity | 0.99  0.88 |
| Oxoxylopine | 450 | 3 | Immunotoxicity  Mutagenicity  Cytotoxicity | 0.99  0.93  0.70 |
| Oxymatrine | 410 | 4 | - | - |
| Oxysanguinarine | 1000 | 4 | Immunotoxicity | 0.84 |
| Pallidine | 400 | 4 | Immunotoxicity | 0.99 |
| Palmatine | 200 | 3 | Immunotoxicity  Mitochondrial membrane potential (MMP) | 0.96  0.84 |
| Palmatrubine | 200 | 3 | Immunotoxicity | 0.91 |
| Papaverine | 69 | 3 | hepatotoxicity | 0.73 |
| Pegaharine A | 1500 | 4 | - | - |
| Pegaharine B | 532 | 4 | - | - |
| Pegaharine C | 560 | 4 | - | - |
| Pegaharine D | 450 | 4 | Immunotoxicity | 0.99 |
| Pegaharine E | 445 | 4 | - | - |
| Pegaharine F | 5000 | 5 | Immunotoxicity | 0.77 |
| Peimisine | 2573 | 5 | Immunotoxicity | 0.81 |
| Perlolyrine | 600 | 4 | mutagenicity | 0.78 |
| Phanostenine | 450 | 4 | Immunotoxicity | 0.99 |
| Physostigima | 1050 | 1 | - | - |
| Pimentelamines A | 4000 | 5 | Immunotoxicity | 0.98 |
| Pimentelamines B | 4000 | 5 | Immunotoxicity | 0.98 |
| Pimentelamines C | 4100 | 5 | Immunotoxicity | 0.99 |
| Piperine | 330 | 4 | Immunotoxicity  Aryl hydrocarbon receptor(AhR)  Estrogen Receptor Alpha(ER)  ATPase family AAA domain containing 5(ATADS) | 0.96  0.99  0.99  1.0 |
| Polycarpine | 350 | 4 | Immunotoxicity | 0.93 |
| Prafumine | 940 | 4 | Immunotoxicity | 0.99 |
| Pronuciferine | 530 | 4 | Immunotoxicity | 0.99 |
| Protopine | 940 | 4 | – | – |
| Pseudoconitine | 41 | 2 | Immunotoxicity | 0.77 |
| Pseudopalmatine | 200 | 3 | Immunotoxicity  Mitochondrial membrane potential (MMP) | 0.96  0.84 |
| Psychotridine | 15 | 2 | - | - |
| Quindolinone | 260 | 3 | - | - |
| Reserpine | 300 | 3 | Carcinogenicity  Immunotoxicity  Nr_ahr  nr_aromatase  sr_mmp | 0.80  0.99  0.99  1.0  0.99 |
| Reticuline | 700 | 4 | Immunotoxicity | 0.92 |
| Roemerine | 450 | 4 | Immunotoxicity | 0.98 |
| Romeline | 450 | 4 | Immunotoxicity | 0.99 |
| Romerine | 450 | 4 | Immunotoxicity | 0.98 |
| Rotundine | 580 | 4 | Immunotoxicity | 0.76 |
| Salsoline | 1000 | 4 | Immunotoxicity | 0.89 |
| Salutaridine | 400 | 4 | Immunotoxicity | 0.98 |
| Sanguinarine | 778 | 4 | Immunotoxicity  Aryl hydrogen Receptor  Estrogen receptor ligand-binding domain  Nuclear factor (erythroid-derived 2)-like 2/ antioxidant responsive elements (nrf2/ARE)  Heat shock factor response element (HSE) | 0.98  1.0  1.0  1.0  1.0 |
| Sanguinine | 85 | 3 | Immunotoxicity  Aryl hydrocarbon receptor | 0.99  0.73 |
| Scopolamine | 1275 | 4 | – | – |
| Securinine | 75 | 3 | - | - |
| Securitinine | 270 | 3 | Immunotoxicity | 0.85 |
| Senbusine A | 28 | 2 | Immunotoxicity | 0.99 |
| Senbusine A | 28 | 2 | Immunotoxicity | 0.99 |
| Simplicifolianine | 440 | 4 | Immunotoxicity | 0.99 |
| Sinomenine | 580 | 4 | - | - |
| Sophocarpine | 196 | 3 | - | - |
| Sophoranol | 410 | 4 | - | - |
| Sophoridine | 243 | 3 | - | - |
| Sophtonseedline A | 410 | 4 | - | - |
| Sophtonseedline B | 418 | 4 | Immunotoxicity | 0.78 |
| Sophtonseedline C | 270 | 3 | Immunotoxicity | 0.89 |
| Sophtonseedline D | 418 | 4 | Immunotoxicity | 0.96 |
| Sophtonseedline E | 500 | 4 | - | - |
| Sophtonseedline F | 1770 | 4 | Immunotoxicity | 0.69 |
| Sophtonseedline G | 410 | 4 | - | - |
| Sophtonseedline H | 1770 | 4 | - | - |
| Sophtonseedline I | 10000 | 6 | - | - |
| Sophtonseedline J | 5000 | 5 | - | - |
| Sophtonseedline K | 5000 | 5 | Immunotoxicity | 0.87 |
| Stepharine | 89470 | 6 | Immunotoxicity | 0.99 |
| Stepharine | 530 | 4 | Immunotoxicity | 0.99 |
| Stepharotudine | 480 | 4 | - | - |
| Strychnogucine B | 1 | 1 |  |  |
| Talcarpine | 300 | 3 | Immunotoxicity | 0.96 |
| Tetrahydropalmatine | 580 | 4 | Immunotoxicity | 0.76 |
| Tetrandrine | 1700 | 4 | Immunotoxicity  Mutagenicity | 0.99  0.84 |
| Thalifoline | 500 | 4 | - | - |
| Thebaine | 54 | 3 | immunotoxicity | 0.98 |
| Trigonelline | 3720 | 5 | - | - |
| Tuduranine | 450 | 4 | Immunotoxicity | 0.97 |
| Tylophoridicine D | 1000 | 4 | Immunotoxicity | 0.97 |
| Undulatine N-oxide | 450 | 4 | immunotoxicity | 0.98 |
| Vasicine | 290 | 3 | - | - |
| Vasicinone | 1100 | 4 | - | - |
| Verticine | 3500 | 5 | Immunotoxicity | 0.87 |
| Verticinone | 280 | 3 | Immunotoxicity | 0.98 |
| Vinblastine | 68 | 2 | Immunotoxicity  cytotoxicity | 0.99  0.96 |
| Vincamajine | 208 | 3 | Immunotoxicity | 0.87 |
| Vincorine | 2 | 1 | – | – |
| Vincristine | 68 | 2 | Immunotoxicity  cytotoxicity | 0.99  0.94 |
| Vindolicine | 150 | 3 | Immunotoxicity | 0.99 |
| Vindolidine | 1 | 1 | - | - |
| Vindoline | 150 | 3 | Immunotoxicity | 0.97 |
| Vindolinine | 325 | 4 | - | - |
| Vireakine | 450 | 4 | Immunotoxicity  Mutagenicity | 0.99  0.74 |
| Virosinine A | 85 | 3 | ATPase family AAA domain-containing protein 5 | 1 |
| Vittacarboline | 380 | 4 | - | - |
| Voacangine | 40 | 2 | Immunotoxicity | 0.99 |
| Xylopinine | 2803 | 5 | - | - |
| α-hydrastine | 1000 | 4 | Immunotoxicity  Aryl hydrogen receptor | 0.99  1 |
| (-)-Norsecurinine | 2573 | 5 | - | - |
| (-)-Thaicanine N-oxide(8,4 hydoxy corynoxidine) | 928 | 4 | Immunotoxicity | 0.82 |
| (+)- norboldine | 450 | 4 | Immunotoxicity | 0.97 |
| (+)-5,6-dehydrolycorine | 230 | 3 | Immunotoxicity | 0.99 |
| (+)-8,9-methylenedioxyl-homolycorine-N-oxide | 230 | 3 | Immunotoxicity | 0.97 |
| (+)-boldine | 450 | 4 | Immunotoxicity | 0.99 |
| (+)-homolycorine-N-oxide | 561 | 4 | Immunotoxicity | 0.79 |
| (+)-laurotetanine | 450 | 4 | Immunotoxicity | 0.98 |
| (+)-N-methyllaurotetanine | 350 | 4 | Immunotoxicity | 0.99 |
| (+)-norboldine acetate | 401 | 4 | Immunotoxicity | 0.99 |
| (+)-reticuline | 700 | 4 | Immunotoxicity | 0.92 |
| (1R, 1S)- N- formylcepharanthine | 750 | 4 | - | - |
| (6S, 7S, 9R, 13S) -7-O- acetyl- N- formylsinococuline | 1900 | 4 | Immunotoxicity  Mutagenicity | 0.99  0.70 |
| (6S, 7S, 9R, 13S)-6, 7-di-O- acetyl- N- formylsinococuline | 400 | 4 | Immunotoxicity | 0.99 |
| (6S, 7S, 9R, 13S)N- formylsinococuline | 400 | 4 | Immunotoxicity | 0.95 |
| .lycoranine A | 1000 | 4 | Immunotoxicity | 0.99 |
| 10-Demethoxyvincorine | 325 | 4 | – | – |
| 10-Demethoxyvincorine N(4)-oxide | 325 | 4 | – | – |
| 10-demethylxylopinine | 2803 | 5 | - | - |
| 11-Demethoxyquaternine | 1190 | 4 | Immunotoxicity.  Aromatase,  Estrogen Receptor Alpha (ER),  Estrogen Receptor Ligand Binding Domain (ER-LBD) | 0.96  1.0  0.99  1.0 |
| 11-methoxyquindoline | 495 | 4 | Mutagenicity | 0.76 |
| 11-Methoxyvincorine | 2 | 1 | – | – |
| 15α-Butoxy-14,15-dihydronorsecurinine | 270 | 3 | Immunotoxicity | 0.82 |
| 15β-Butoxy-14,15-dihydronorsecurinine | 270 | 3 | Immunotoxicity | 0.82 |
| 19,20-Z-Affinisine | 760 | 4 | – | – |
| 20,21-Dihydroalstonerine | 300 | 3 | Immunotoxicity | 0.77 |
| 2α-methoxy-6-O-ethyloduline | 230 | 3 | Immunotoxicity | 0.96 |
| 3’-nor-4’- oxocepharanthine | 1900 | 4 | Immunotoxicity | 0.99 |
| 4-epiphyllanthine | 270 | 3 | Immunotoxicity | 0.85 |
| 5.6-dehydro-matrine | 418 | 4 | - | - |
| 5-hydroxy-6.7-dehydro-matrine | 480 | 4 | - | - |
| 5α- hydroxysophocarpine | 418 | 4 | - | - |
| 5α-9α-dihydroxymatrine | 410 | 4 | - | - |
| 6.7- dehydro-matrine | 480 | 4 | - | - |
| 6a-methoxycrinamidine | 450 | 4 | Immunotoxicity | 0.99 |
| 6-hydroxycrinamine | 86 | 3 | Immunotoxicity  Cytotoxicity | 0.99  0.71 |
| 6-methoxydihydrosanguinarine | 2000 | 4 | Immunotoxicity,  Mutagenicity | 0.98  0.72 |
| 6S, 7S, 9R, 13S-6-O-acetyl-N-formylsinnococuline | 28 | 2 | Immunotoxicity | 0.99 |
| 7a-(pyridine-2-yl)-7,7adihydrofuran-2(6H) | 12 | 2 | - | - |
| 8-hydoxyquinoline | 280 | 3 | -Nuclear factor  (erythroid-derived 2)-like 2/antioxidant responsive element(nrf2/ARE)  -Heat shock factor response element (HSE)  -Phosphoprotein (tumor suppressor)p53 | 1.0  1.0  1.0 |
| 9α-hydroxymatrine | 410 | 4 | - | - |
| 9α-hydroxysophocarpine | 418 | 4 | - | - |

**Figure S1** Molecule structure of plant-derived alkaloids

**Figure S1** Continued

**Figure S1** Continued

**Figure S1** Continued

**Figure S1** Continued

7 **Figure S1** Continued

**Figure S1** Continued

 **Figure S1**
